# Rationally designed self-assembled peptide nanofibers provoke robust humoral immunity against nervous necrosis virus

**DOI:** 10.1128/jvi.00319-25

**Published:** 2025-07-15

**Authors:** Chen Zhang, Yong-Can Zhou, Wen-Ye Song, Xin-Xin Liu, Hai-Hua Peng, Yun Sun

**Affiliations:** 1 School of Marine Biology and Fisheries, Sanya Institute of Breeding and Multiplication, Collaborative Innovation Center of Marine Science and Technology, Hainan University 74629, Haikou, China; St. Jude Children's Research Hospital, Memphis, Tennessee, USA

**Keywords:** phage display technology, antigenic epitope, vaccine design, modular peptide vaccine, humoral immunity, nervous necrosis virus

## Abstract

Nervous necrosis virus (NNV) poses devastating mortality risks to almost all marine fish, causing significant economic losses to the marine fish aquaculture industry, particularly in juvenile groupers. However, no effective NNV vaccine is available. To counter this, we report a smart strategy of a rationally designed modular peptide vaccine platform (CP^64-83^-Q11) that recapitulates the antigen peptide CP^64-83^ and a Q11 self-assembling domain. An anti-NNV phage antibody library was constructed and used for screening anti-NNV antibody. Bioinformatics and surface-plasmon resonance were utilized to identify the antigen epitope of NNV. Furthermore, the CP^64-83^-Q11 nanofiber was designed and constructed. The cellular and tissular uptake of CP^64-83^-Q11 was analyzed i*n vitro* and *in vivo*. Moreover, the immunogenicity and the protective efficacy of the CP^64-83^-Q11 nanofibers were evaluated by determining the expression of antigen-presenting indicators, antibody levels, and response to viral challenge to vaccinated fish. It has been proven that CP^64-83^-Q11 nanofiber could efficiently deliver antigen peptide to immune tissues and induce robust humoral immunity against NNV infection, which provided a protection rate of approximately 88%. This study provides an effective strategy for aquatic vaccine design, which will be beneficial for the application and development of vaccines in the aquaculture industry.

**IMPORTANCE:**

Viral diseases have constantly caused a great threat to global public health, resulting in an urgent need for effective vaccines. However, the current viral vaccines are often showing low immunogenicity. NNV, a serious threat to almost marine fish, was used as a viral model in this study. To address the threat of NNV, an anti-NNV antibody phage library was constructed and used for anti-NNV antibody biopanning. Based on bioinformatics and surface-plasmon resonance, we validated an antigen peptide for NNV (CP^64-83^) with high-affinity binding ability to the anti-NNV antibody. Moreover, a modular peptide vaccine delivery platform (CP^64-83^-Q11) was rationally designed using phage display technology and bioinformatics. CP^64-83^-Q11 could efficiently deliver into immune tissues and provide effective protection against NNV infection by inducing a significant level of humoral immunity. This study offers new insights into rational viral vaccine design for fish, which could provide methods for the prevention and treatment of animal viral diseases.

## INTRODUCTION

Viruses have long been recognized as formidable threats to global health, causing a wide array of diseases ranging from mild infections to life-threatening conditions (1, 2). Among the numerous viruses, nervous necrosis virus (NNV) has emerged as a significant concern, especially in the aquaculture industry (3). NNV, belonging to the genus *Betanodavirus* and family Nodaviridae, is a pathogen that primarily affects fish, causing severe neurological symptoms, such as abnormal swimming behavior, loss of appetite, and high mortality rates (4). This virus can infect a wide variety of fish species, including important commercial species, such as sea bass, sea bream, and especially grouper (5). Furthermore, NNV infection causes high lethality in juvenile fish, with mortality rates reaching 100% (6). The economic impact of NNV is substantial, as it not only leads to direct losses in fish production but also requires significant investment in disease control measures. To date, no licensed vaccines or specific therapeutics are available for NNV prevention or treatment.

As the oldest vertebrates, teleost fish possess a relatively complete immune system, featuring both innate and adaptive immune responses (7). When the teleost fish are infected with NNV, the immune system recognizes these antigenic proteins as foreign substances and initiates an immune response. Based on this principle, researchers have developed various vaccines to prevent NNV infections (8). These vaccines typically contain inactivated or attenuated viruses or their antigenic components. However, despite continuous efforts in vaccine development, the existing NNV vaccines have shown relatively weak immune effects, high potential safety risks, or high production costs (9).

One promising approach to improve the immunogenicity of NNV vaccines is through the screening of dominant antigenic epitopes of the virus. Antigenic epitopes are specific regions on an antigen molecule that are recognized by the immune system (10). By identifying and incorporating the most immunogenic epitopes into vaccines, we can enhance the ability of vaccines to stimulate a robust immune response (11). NNV possesses only one structural protein (capsid protein, CP), which serves as the primary immunogen and plays critical roles in viral assembly, diagnostic detection, and host specificity, making it a key target for antiviral research (12). The current NNV vaccines typically employ a relatively complete CP protein as the antigen. However, the CP protein also contains some redundant proteins that do not have antigenic activity. The specific antigenic epitopes of CP protein remain unclear. Phage display technology offers several distinct advantages in the field of antigenic epitope screening (13). Firstly, it is a highly versatile and high-throughput technique (14). A vast library of peptides can be displayed on the surface of bacteriophages, allowing for the rapid screening of millions of potential epitopes (15, 16). This large-scale screening capability significantly increases the chances of identifying the most immunogenic epitopes (17). Secondly, phage display technology is relatively simple and cost-effective compared to other traditional epitope mapping methods (18). It does not require complex and expensive equipment, making it accessible to a wide range of research laboratories (19). In recent years, phage display technology has been widely applied in antigenic epitope screening for various viruses (20). For example, in the study of influenza virus, researchers have used phage display technology to identify novel antigenic epitopes that can be used as components of next-generation vaccines (21). These identified epitopes can potentially overcome the limitations of current influenza vaccines, such as the need for annual updates due to antigenic drift (22). In the case of NNV, phage display technology can also be exploited to screen for dominant antigenic epitopes on the virus’s antigen proteins. By isolating and utilizing these epitopes in vaccine development, we can potentially improve the immune effect of NNV vaccines, providing better protection for fish in aquaculture.

In addition to screening for dominant antigenic epitopes, enhancing the delivery efficiency of vaccines is another crucial method in improving their immunogenicity (23, 24). Efficient vaccine delivery ensures that the antigenic components reach the appropriate immune cells in a timely and effective manner, thus maximizing the immune response (25, 26). Poor delivery can result in low antigen uptake by immune cells and limited induction of immune responses (27). Nanomaterials have shown great potential in enhancing vaccine delivery efficiency (28). They possess several inherent advantages, such as their small size, high surface-to-volume ratio, and the ability to be engineered with specific functionalities (29, 30). Due to their small size, nanomaterials can easily penetrate biological barriers, including cell membranes, and deliver antigens directly to the cytoplasm of immune cells (31). Their high surface-to-volume ratio allows for a large amount of antigen to be loaded onto the nanomaterial surface (32). Additionally, nanomaterials can be modified with targeting ligands to specifically deliver antigens to certain types of immune cells, such as dendritic cells, which play a key role in initiating immune responses (33). Among the various nanomaterials, Q11 self-assembling peptides have unique advantages in short-peptide vaccine delivery (34). Q11 is a short peptide that can spontaneously assemble into stable nanostructures under physiological conditions (35). These self-assembled nanostructures can encapsulate short-peptide antigens, protecting them from degradation in the biological environment (36). Moreover, the surface properties of Q11 self-assembled nanostructures can be easily tuned to enhance the interaction between the antigens and the immune cells (37). For example, by introducing specific functional groups on the surface of the nanostructures, we can increase the uptake of antigens by immune cells.

In this study, we report a rationally designed modular peptide vaccine platform (CP^64-83^-Q11) against nervous necrosis virus, a serious and mortal threat to almost marine fish. To counter the threat of NNV, an anti-NNV antibody phage library was constructed. Using the specific phage library, high-affinity specific scFv (A2) was screened. Furthermore, CP^64-83^ was validated using bioinformatics and surface-plasmon resonance, which showed high-affinity binding with A2. CP^64-83^ was combined with a self-assembling domain Q11 to develop a nanofiber (CP^64-83^-Q11), which confers excellent antigen delivery ability. CP^64-83^-Q11 has been proven to be able to induce robust humoral immunity and high immunoprotection. Our findings provide a new strategy and reference for constructing efficient peptide vaccines against fish viral diseases.

## RESULTS

### Construction and biopanning of the anti-NNV phage display antibody library

**Fig 1:**
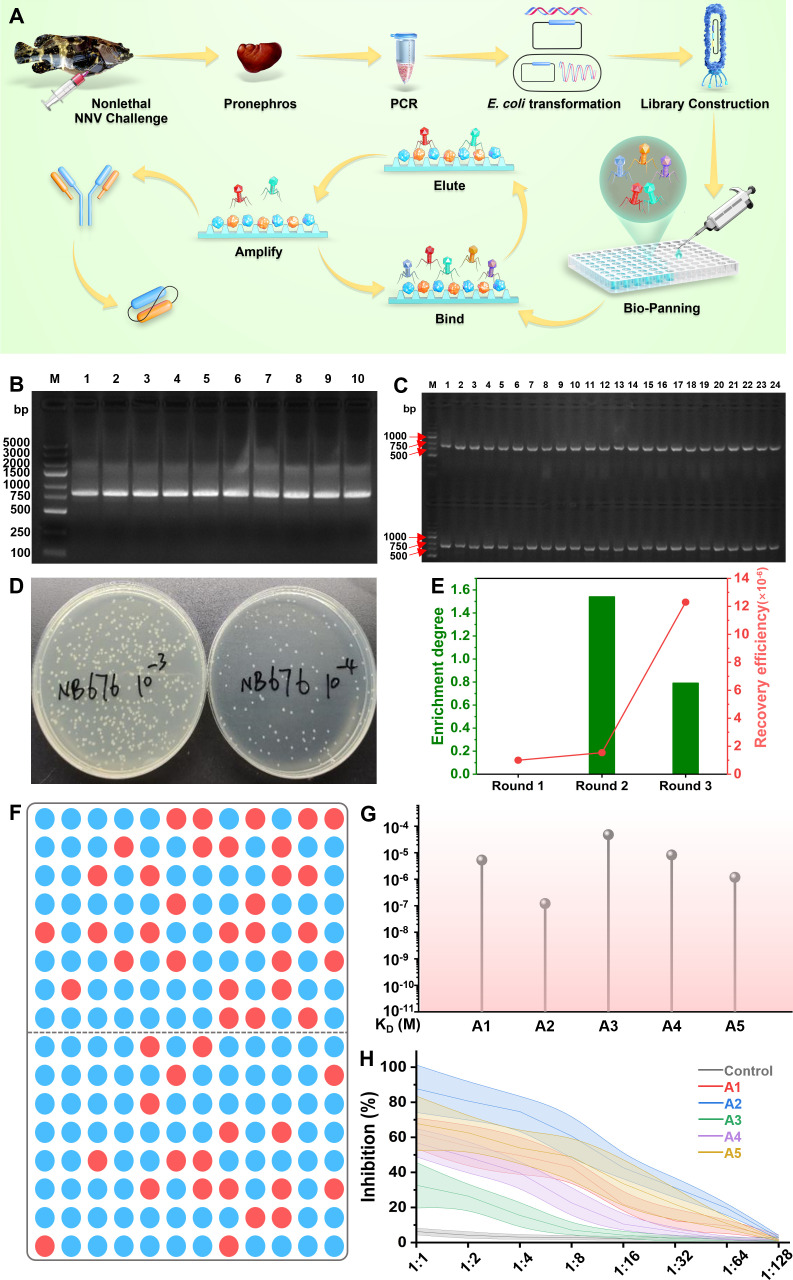
Construction and biopanning of the anti-NNV phage display antibody library. (**A**) Schematic diagram of the construction and biopanning strategy for anti-NNV-specific single-chain antibody fragment (scFv). The purified NNV was used as the target, and the immunopositive phage clones were further identified through sequencing and enzyme-linked immunosorbent assay (ELISA) after three rounds of biopanning. (**B**) The electrophoresis results of scFv. (**C**) Colony PCR analysis to identify the insertion rates of VHH fragment. (**D**) Tittering of phage of tenfold serial dilutions. (**E**) Enrichment degree and recovery efficiency of biopanning for anti-NNV phage display antibody library. (**F**) Distribution diagram of positive clones after the third round of panning, where red represents positive clones and blue represents a negative clone. (**G**) The affinity of scFv analyzed by SPR assay. (**H**) Affinity of purified scFv’s to NNV detected by indirect ELISA.

As shown in Fig. 1A, healthy groupers are intraperitoneally injected with nonlethal NNV. On the fourth week post-challenge, the spleen of the vaccinated fish was isolated and used to extract RNA. Figure S1 indicates that the grouper spleen RNA shows high purity (A260/A280 = 2.03, A260/A230 = 2.17). After three rounds of nested PCR, amplified *V_H_* and *V_L_* genes were cloned with the expected product size (approximately 500 bp) (Fig. 1B; Fig. S2 and S3), which was consistent with the theoretical value of heavy/light strand base number. A total of 100 mL resuscitation product was obtained after 10 cycles of electric shock transformation. The resuscitation product was incubated at 37°C for 45 min. Then, 100 µL of the product was serially diluted to 10^−3^ and 10^−4^ to determine the number of converters in the anti-NNV antibody library. On the second day, there was an average of 149 clones on the 10^−4^ plate (Fig. 1D); therefore, the constructed anti-NNV library had a number of transformants of 1.49 × 10^9^ cfu.

After three rounds of biopanning, the enrichment degree and recovery efficiency in each round are shown in Fig. 1E and Table S2. The recovery efficiencies (output/input ratios) of three rounds were 1.00 × 10^−6^, 1.54 × 10^−6^, and 1.23 × 10^−5^, respectively. However, the enrichment degrees were reduced, which were 1.54 and 0.79 among three rounds of biopanning, respectively. One hundred ninety-two phage monoclones were randomly selected from the third round of biopanning, and 50 positive clones were detected (Fig. 1F). Among these positive clones, 41 clones were successfully sequenced. Considering the sequence similarity of scFv, six antibodies (A1, A2, A3, A4, A5, and A6) with the highest affinity were selected for protein preparation. The affinity ability between NNV and scFv was further analyzed by surface-plasmon resonance (SPR). The A2 group showed the lowest dissociation constant (*K*_*D*_), which indicates the strongest affinity ability (Fig. 1G). As shown in Fig. 1H, significantly, the strongest NNV-neutralizing activity at 28 dpv is observed in the A2 group among these six scFv. No inhibition ability was detected in the control group.

### High-affinity antibody identification and antigen–antibody interaction site bioinformatics analysis

**Fig 2:**
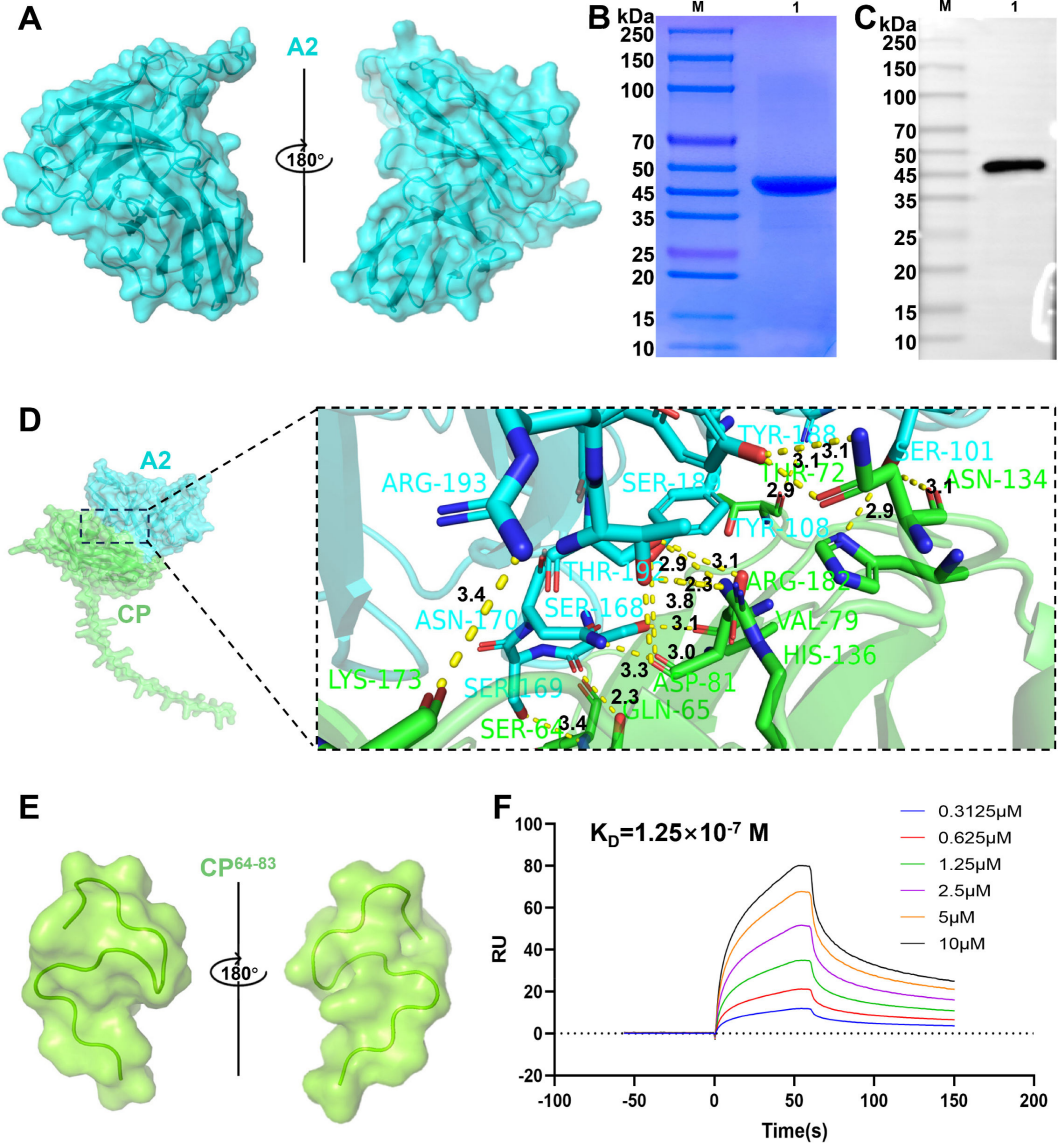
High-affinity antibody identification and antigen–antibody interaction sites bioinformatics analysis. (**A**) Tertiary structure of A2 predicted by AlphaFold3 and analyzed using PyMOL. (**B**) SDS-PAGE analysis of A2 protein. Lane M: standard protein marker; Lane 1: purified A2 protein. (**C**) Western blotting analysis of purified A2 protein. Lane M: standard protein marker; Lane 1: purified A2 protein. (**D**) The molecular docking results of the A2-CP complex. A2 is shown in blue, and CP is shown in green. Close-up views of the A2-occupied sites in CP are displayed. The blue ribbons represent A2, the green ribbons represent CP, and the hydrogen bonds are shown as yellow dashes. (**E**) Predicted tertiary structure of CP^64-83^. (**F**) Confirmation of the interaction between A2 protein and CP^64-83^ using surface-plasmon resonance (SPR) method.

The 3D structure of A2 scFv was predicted and is shown in Fig. 2A. The results indicated that purified A2 protein showed an expected molecular weight of approximately 46.8 kDa (Fig. 2B and C). The key amino acid residues for CP-A2 interactions are shown in Fig. 2D and Fig. S4, and the results indicated that the region from the 64th amino acid to the 83^rd^ amino acid of CP protein was more likely to bind A2. The predicted 3D structure of CP^64-83^ is shown in Fig. 2E. The affinity ability between CP^64-83^ and A2 was further evaluated by SPR. As depicted in Fig. 2F, the *K*_*D*_ value of CP^64-83^-A2 is 1.25 × 10^−7^, which indicated the strong affinity ability between CP^64-83^ and A2.

### Construction and characterization of CP^64-83^-Q11 peptide nanofiber

**Fig 3:**
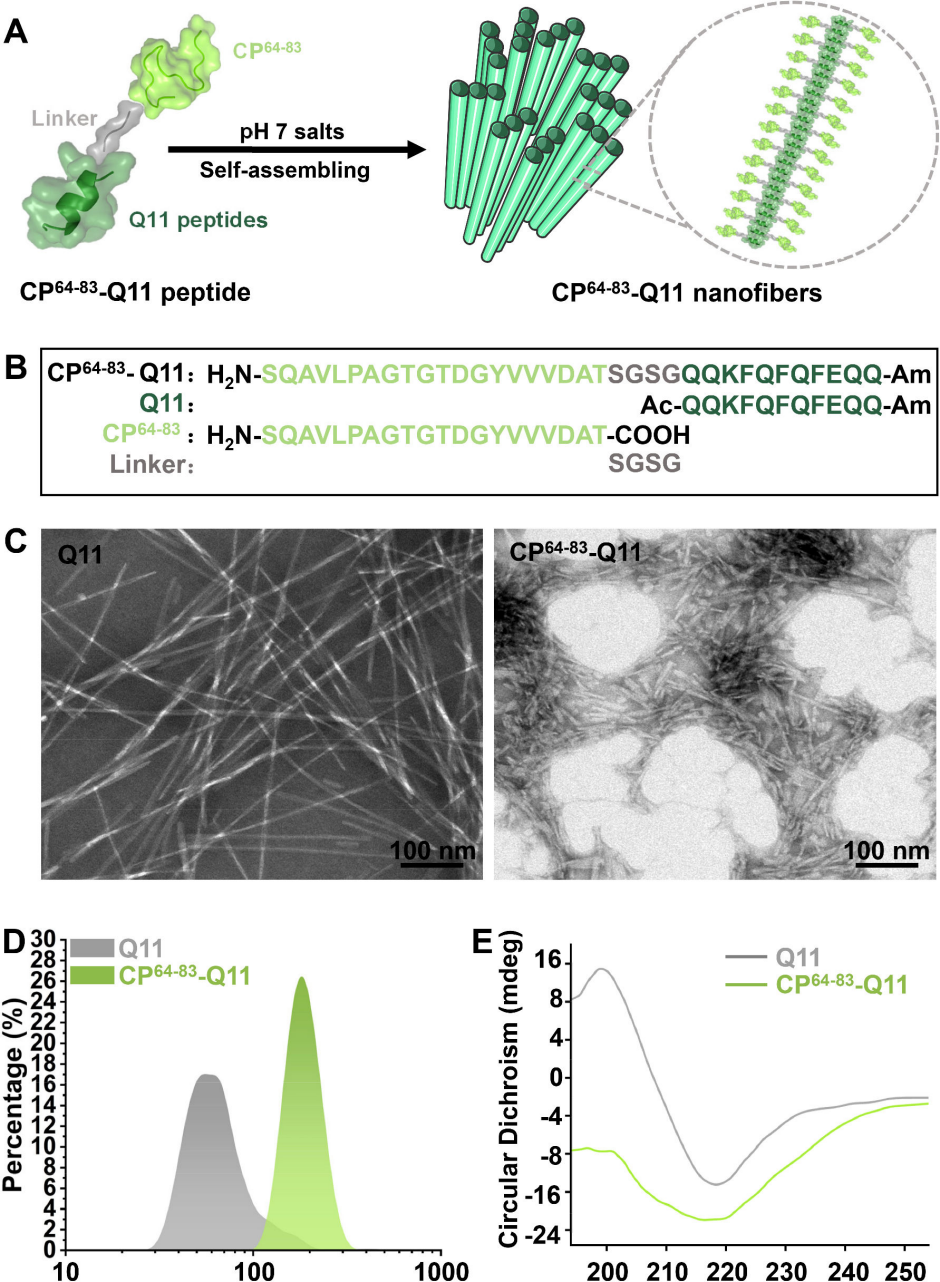
Construction and characterization of CP^64-83^-Q11 peptide nanofibers. (**A**) Schematic illustration showing the construction of CP^64-83^-Q11 peptide nanofibers. (**B**) The protein sequence of CP^64-83^-Q11 peptide. (**C**) The transmission electron microscopy (TEM) results of CP^64-83^-Q11 peptide nanofibers. (**D**) Hydrodynamic size of CP^64-83^-Q11 peptide nanofibers. (**E**) The circular dichroism spectrum results of CP^64-83^-Q11 peptide nanofibers.

The interaction of vaccines and TLRs is an important indication for proper protection against pathogens. We firstly predicted the interactions between CP^64-83^ and TLRs. Figure S5 showed that CP^64-83^ had a high possibility of interacting with TLR1, TLR2, TLR3, and TLR7. Therefore, we choose CP^64-83^ as the antigen for the construction of the NNV vaccine. In this study, we designed a modular peptide delivery platform containing a Q11 self-assembling domain in tandem with CP^64-83^, a peptide from antigen protein CP of NNV (Fig. 3A and 1B). We then characterized the developed CP^64-83^-Q11 nanofiber. Transmission electron microscopy (TEM) revealed a uniform nanofiber structure of CP^64-83^-Q11 (Fig. 3C). Figure 3D shows the size of the CP^64-83^-Q11 nanofiber. CP^64-83^-Q11 exhibited an average size of 187.6 nm. The circular dichroism (CD) spectra showed that CP^64-83^-Q11 nanofiber showed typical β-sheet characteristics (Fig. 3E). The above results indicated that a self-assembling nanofiber was successfully constructed.

We further evaluated the biosafety of CP^64-83^-Q11 *in vitro* and *in vivo*. The potential cytotoxicity of CP^64-83^-Q11 toward GF-1 cells and macrophages was determined by 3-(4,5-dimethyl-2-thiazolyl)-2,5-diphenyl-2-H-tetrazolium bromide and thiazolyl blue tetrazolium bromide (MTT) assays, respectively. CP^64-83^-Q11 did not affect cytotoxicity even at a high vaccine concentration (40 µg/mL) (Fig. S6). Moreover, as shown in Table S3, there are no significant differences in survival and weight between immunized and control groupers. Meanwhile, no obvious pathological change was observed in fish tissues (gill, liver, kidney, spleen, intestine, and brain) after vaccination. The above results implied good biosafety of the constructed CP^64-83^-Q11 nanofiber.

### Uptake of FITC-labeled CP^64-83^-Q11 nanofiber *in vitro* and *in vivo*

**Fig 4:**
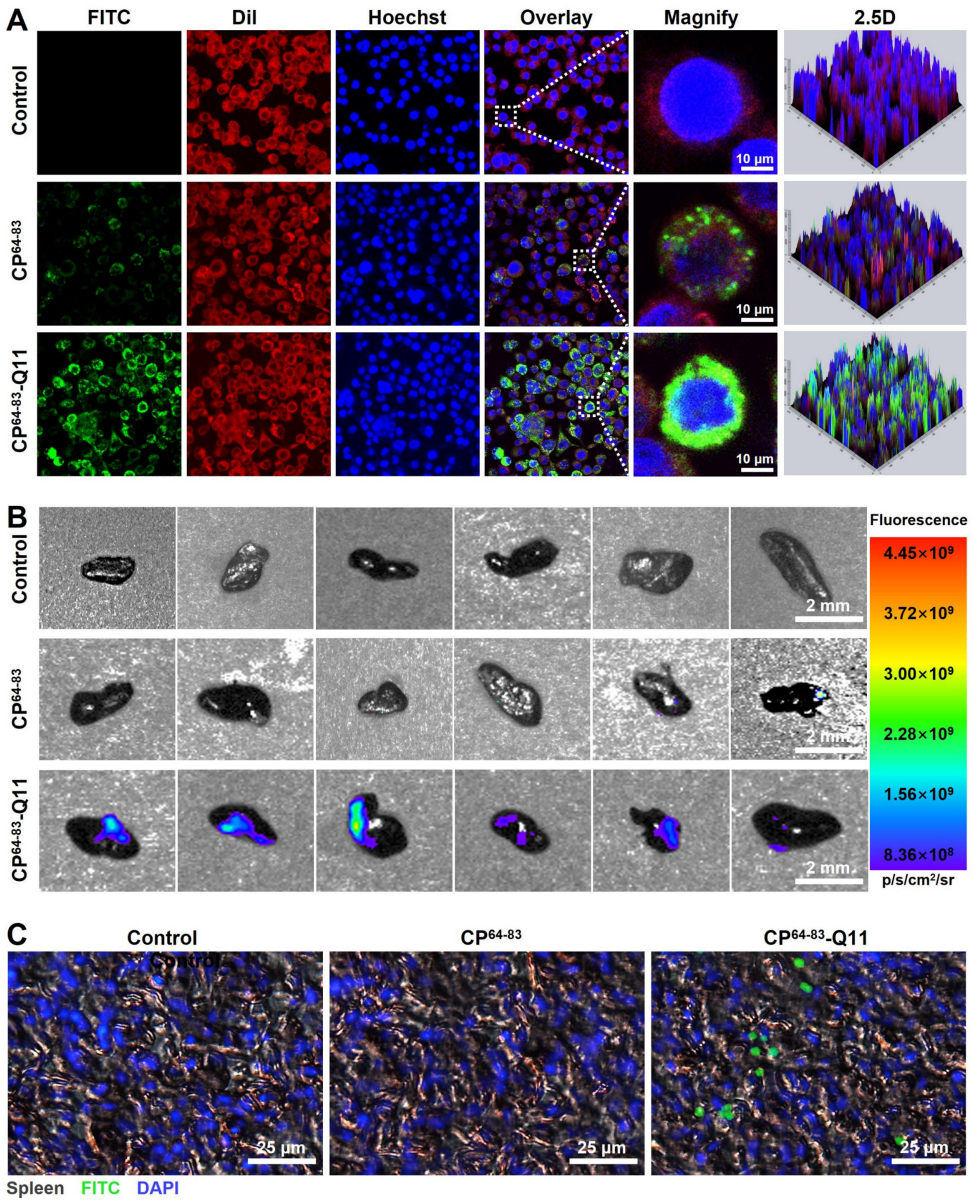
Uptake of FITC-labelled CP^64-83^-Q11 nanofibers *in vitro* and *in vivo*. (**A**) Representative confocal laser scanning microscope images of macrophages treated with FITC-labeled CP^64-83^-Q11 nanofibers. (**B**) Representative *ex vivo* fluorescence images of the isolated spleen after immunization of FITC-labeled CP^64-83^-Q11 nanofibers. (**C**) Representative immunofluorescence images of the spleen dissected from the fish injected with FITC-labeled CP^64-83^-Q11 nanofibers.

The cellular and tissular uptake of CP^64-83^-Q11 nanofiber was evaluated. GF-1 cell was used as a cell model. Confocal laser scanning microscope (CLSM) and flow cytometry were used to analyze the cellular uptake of CP^64-83^-Q11 nanofiber. As depicted in Fig. 4A, it can be observed that the CP^64-83^ group showed similar fluorescence intensity with the control group. Notably, the CP^64-83^-Q11 group showed stronger green fluorescence than the control and CP^64-83^ groups. The results of flow cytometry showed a similar phenomenon: the mean fluorescence intensity in the CP^64-83^-Q11 group was the strongest, which was significantly higher than that in the CP^64-83^ and control groups (Fig. S7). The uptake of CP^64-83^-Q11 nanofiber in fish spleen was analyzed. As shown in Fig. 4B, the CP^64-83^-Q11 group shows obvious fluorescence signal; however, the control and CP^64-83^ groups show barely detectable fluorescence signal. The results of fluorescence intensity indicated that the mean fluorescence intensity in the CP^64-83^-Q11 group was significantly higher than that in CP^64-83^ groups (Fig. S8). The tissular uptake of CP^64-83^-Q11 nanofiber was further evaluated by CLSM. As expected, an obvious green fluorescence signal was observed in the CP^64-83^-Q11 group (Fig. 4C; Fig. S9).

### Evaluation of the adaptive immune responses induced by CP^64-83^-Q11 nanofiber

**Fig 5:**
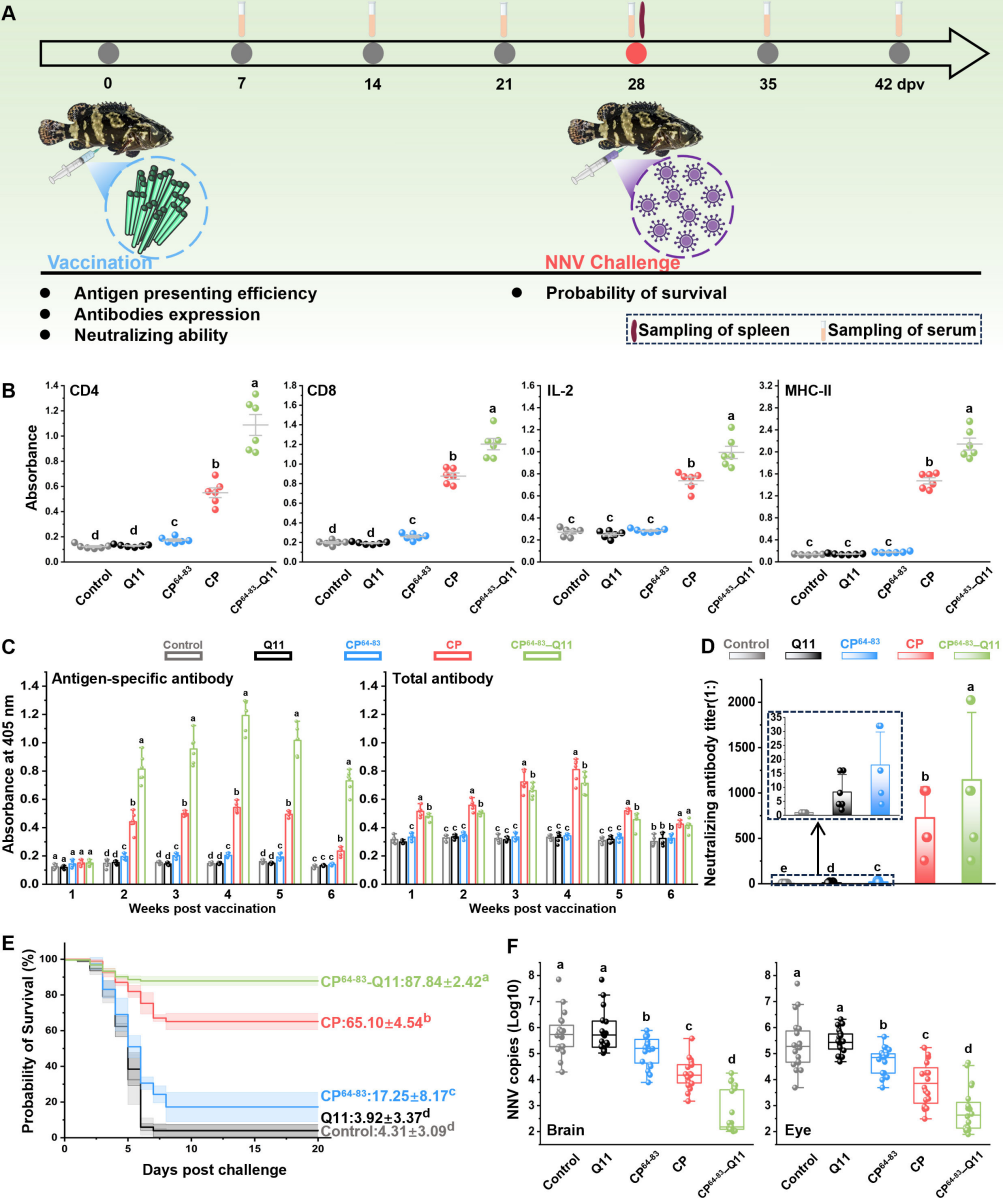
Evaluation of the adaptive immune responses induced by CP^64-80^-Q11 nanofibers. (**A**) Schematic diagram of the immunization and challenge experiments. (**B**) Expression of antigen-specific antibodies and total antibodies in serum from weeks one to six post-vaccination. (**C**) Neutralizing antibodies on the fourth week after vaccination. (**D**) Expression of CD4, CD8, IL-2, and MHC-II in different groups. (**E**) Survival rate of fish after NNV challenge. (**F**) Number of virus copies in the brain and eye on the eighth day post-challenge. The *P*-value in (**B–E**) was determined by Student’s *t*-tests. *P* values in (**E**) were calculated by Log-rank (Mantel-Cox) Test. Data at the same sampling time with different lowercase letters (**a, b, c, d, and e**) are significantly different (*P* < 0.05).

The experiment design and sampling strategy are illustrated in Fig. 5A. As shown in Fig. 5B, vaccination significantly induces the protein expression levels of CD4, CD8, IL-2, and MHC*-*II. Notably, the expression levels of these indicators in CP^64-83^-Q11 group were significantly higher than those in the CP group. After immunization, the levels of antigen-specific antibodies and total antibodies in the experimental group increased with the duration of immunization, peaked at the fourth week, and then decreased. These results showed that vaccination of both CP and CP^64-83^-Q11 induces robust antibodies in grouper serum. Importantly, the expression level of antibodies in the CP^64-83^-Q11 group was significantly higher than in the CP group (Fig. 5C). The neutralizing antibody titers in serum at the fourth week after immunization are shown in Fig. 5D. The vaccinated groups showed significantly higher neutralizing antibody titers; moreover, the CP^64-83^-Q11 group showed the highest neutralizing antibody titers, followed by the CP, CP^64-83^, Q11, and control groups. As depicted in Fig. 5E, the survival rates of the control group and Q11 group are approximately 4.31% and approximately 3.92%, respectively. The survival rate of the CP^64-83^-Q11 group was the highest, which was approximately. 87.84%, which was significantly higher than the survival rate of approximately 65.10% in the CP group. The number of virus copies in the brain and eye of the control group was the highest on the eighth day after the challenge, followed by the Q11 group, the CP^64-83^ group, and the CP group, with the CP^64-83^-Q11 group showing the lowest number (Fig. 5F).

## DISCUSSION

Vaccination represents a key strategy for disease prevention and control in aquaculture. However, achieving effective immunization remains the primary constraint for current aquaculture vaccines. In this study, using NNV as a model, we employed phage display technology coupled with bioinformatic analysis to screen and identify dominant antigenic epitopes within the NNV capsid protein. We further developed a peptide vaccine delivery platform (CP^64-83^-Q11 nanofiber) based on the self-assembling peptide Q11. Notably, CP^64-83^-Q11 nanofiber elicited robust humoral immune responses and significantly enhanced protective immunity against NNV infection.

Grouper is one of the main infection targets of NNV. The mortality rate of infected juvenile grouper can reach up to 100%. Therefore, we used grouper as the experimental animals to evaluate the immune effect of the constructed vaccine. This study reveals that CP^64-83^-Q11 can evoke a notable elevation in the expression levels of IgM in grouper fish. Specifically, the IgM expression levels in the group treated with CP^64-83^-Q11 are significantly higher than those in the CP group. Moreover, the results of the NNV challenge experiment also show that the immune protection rate of the CP^64-83^-Q11 group was the highest after the NNV infection, which was significantly higher than that of the other immune groups (CP^64-83^ and CP group). These findings indicate that CP^64-83^-Q11 is capable of triggering a robust humoral immune response in grouper. Grouper is a typical teleost fish. Teleost fish, the most ancient bony vertebrates possessing a classical immune system, exhibit both innate and adaptive immunity (38). Similar to mammals, their adaptive responses encompass systemic and mucosal components. Immunoglobulins (Igs) are pivotal mediators of this adaptive defense (39). Unlike mammals, however, teleosts express only three Ig isotypes: IgM, IgT, and IgD. IgM, the dominant circulating antibody analogous to its mammalian counterpart, primarily mediates systemic immunity (40). Conversely, the teleost-specific IgT functions as a specialized mucosal immunoglobulin, mirroring the role of IgA in mammals. The biological function of IgD remains less defined (41). Therefore, the expression level of IgM is one of the important indicators for evaluating the humoral immunity in teleost fish.

We successfully screened the antigenic epitopes of the CP protein using phage display technology and bioinformatics. Phage display technology has revolutionized antigenic epitope identification by enabling high-throughput screening of peptide libraries through the fusion of foreign peptides with phage surface proteins (42, 43). This approach leverages the genetic flexibility of bacteriophages (e.g., T7 or M13) to express billions of random peptide sequences, allowing systematic interrogation of host–pathogen interactions (44). A key advantage lies in its ability to identify conformational and linear epitopes with high specificity, bypassing the limitations of traditional serological methods (45). For instance, this technique has been instrumental in mapping neutralizing epitopes of enveloped viruses like SARS-CoV-2, where receptor-binding domain (RBD)-specific epitopes were rapidly characterized during the pandemic (46). The integration of next-generation sequencing with phage display further enhances epitope discovery efficiency, enabling the identification of rare but immunodominant epitopes that evade conventional detection (47). Applications extend beyond vaccine design to the development of diagnostic tools. Phage-derived epitopes serve as antigenic probes in ELISA and lateral flow assays, improving the accuracy of serosurveillance for emerging viruses (48). Recent advancements in *in vivo* phage display, such as targeting tissue-specific vascular receptors, open avenues for organ-selective antigen delivery (49). Moreover, the technology’s compatibility with synthetic biology—exemplified by engineered phages carrying adjuvant motifs—promises synergistic enhancement of adaptive immunity (50). Future directions include personalized epitope vaccines for highly mutable pathogens (e.g., HIV and influenza) and phage-based platforms for rapid response to zoonotic viral threats, aligning with One Health initiatives (51).

In the context of NNV vaccine development, using Q11 self-assembling peptides to deliver short-peptide antigens derived from dominant antigenic epitopes can significantly improve the presentation efficiency of the vaccine, thereby enhancing the overall immune effect of the vaccine against NNV infections. Due to the unique structure of the nanofibers, CP^64-83^-Q11 can accumulate in large amounts in the immune tissue (Fig. 4). Within a certain range, more antigens enter the immune tissue, and the host will generate a stronger immune response. The Q11 peptide (QQKFQFQFEQQ), a minimalist self-assembling motif, exemplifies the convergence of nanotechnology and immunology in vaccine design. Its β-sheet-driven fibrillization forms stable nanostructures that enhance antigen multivalency and prolong lymph node retention, addressing the poor immunogenicity of short-peptide antigens (52). Unlike traditional adjuvants (e.g., alum), Q11 avoids excessive inflammation while promoting dendritic cell uptake via size-dependent drainage and scavenger receptor-mediated endocytosis (34). Studies on model antigens, such as ovalbumin, demonstrated that Q11 conjugates elicit robust CD4^+^ T-cell and antibody responses, comparable to Freund’s adjuvant but with superior biocompatibility (53). Q11’s modularity enables co-assembly with diverse epitopes and toll-like receptor (TLR) agonists, creating tunable “vaccine cocktails.” For instance, integrating Q11 with SARS-CoV-2 RBD peptides and TLR7/8 ligands induced sterilizing immunity in murine models, highlighting its potential for rapid pandemic preparedness (54). Additionally, its thermostability and absence of cold-chain requirements make it ideal for global distribution—a critical advantage highlighted during COVID-19 vaccine rollout challenges (55). Further optimization, such as protease-resistant D-amino acid substitutions, could extend its utility in mucosal vaccines against respiratory and enteric viruses (52). This combination of potent immunogenicity, safety, and design flexibility makes Q11 highly promising for next-generation subunit vaccines.

The constructed CP^64-83^-Q11 is a peptide vaccine. Peptide vaccines represent a paradigm shift in antiviral prophylaxis, combining synthetic simplicity with precision immunity. Unlike whole-pathogen or protein-based vaccines, peptides avoid allergenic or autoimmune risks by excluding non-protective epitopes (56). Their primary strength resides in exceptional safety profiles, as these nonviable constructs eliminate risks of pathogenicity reversion or genomic integration associated with conventional platforms. Precision antigen design ensures high specificity toward conserved pathogenic determinants while avoiding allergenic or immunosuppressive components often present in whole-pathogen formulations (57). Manufacturing advantages include reproducible chemical synthesis under Good Manufacturing Practice (GMP) conditions, bypassing complex biological production systems and enabling rapid development cycles—particularly valuable against emerging pathogens. Thermostability and design modularity further permit combinatorial incorporation of adjuvant molecules and universal epitopes to broaden immune coverage. However, critical constraints persist, primarily due to inherent immunogenicity limitations (58). Low molecular mass facilitates rapid renal clearance and extracellular degradation, necessitating potent adjuvants or delivery vehicles (e.g., liposomes and polymeric nanoparticles) to enhance dendritic cell uptake and cross-presentation (59). MHC restriction inherently narrows population coverage, demanding HLA-promiscuous epitopes or personalized approaches (60). Consequently, while epitope-focused vaccines represent a rationally engineered platform with superior safety and adaptability, their translational success hinges on overcoming delivery barriers and immunological shortcomings through innovations in self-assembling carriers, nanoparticle encapsulation, and synergistic immune potentiators—advances progressively demonstrated in oncological and infectious disease clinical trials. Q11-based aquatic peptide vaccines offer transformative potential for sustainable aquaculture disease control through precise epitope targeting and eliminated virulence risks, enabling safer application than conventional platforms. Their synthetic production ensures cost-effective scalability against emerging pathogens like NNV. However, realizing commercial viability requires overcoming inherent immunogenicity limitations via advanced adjuvants and MHC restriction barriers through multi-epitope engineering. Future success hinges on optimizing delivery systems for prolonged efficacy across diverse species, positioning these vaccines as pivotal, eco-friendly alternatives to antibiotics in expanding global aquaculture.

**Fig 6:**
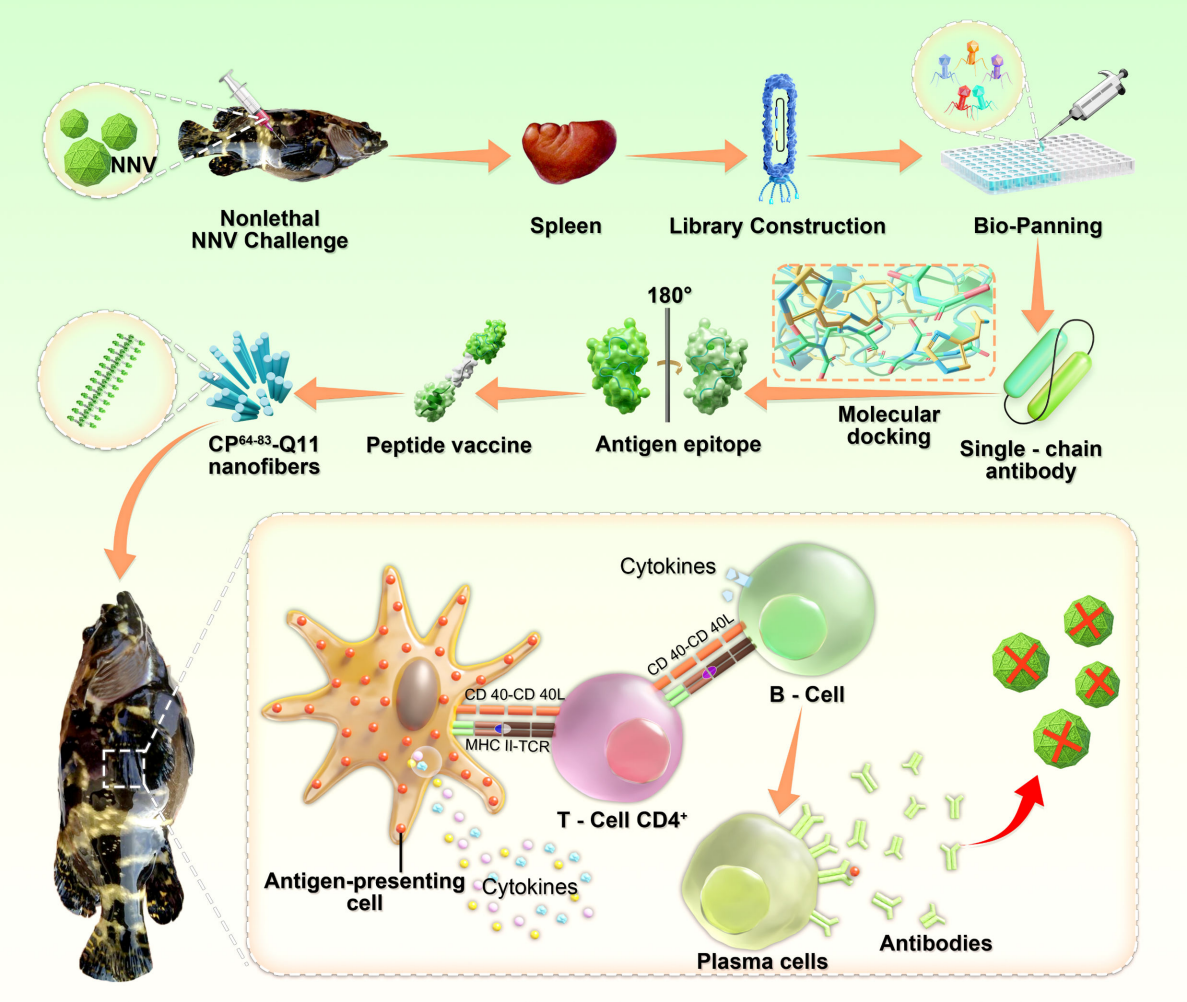
Schematic illustration of the CP^64-80^-Q11 nanofibers design and their function in inducing robust humoral immunity as a vaccine against nervous necrosis virus.

In summary, we reported a peptide vaccine design strategy based on the phage display technology and bioinformatics. NNV was used as a viral model to evaluate the constructed peptide vaccine (CP^64-83^-Q11). It has been proven that CP^64-83^-Q11 could efficiently deliver to immune tissues and induce robust humoral immunity against NNV infection. This study provides a certain reference for antigenic peptide screening and a peptide vaccine design strategy for the prevention and treatment of pathogens (Fig. 6).

## MATERIALS AND METHODS

### Virus and animals

NNV (strain HN508, genotype RGNNV, accession number: PP066840.1), isolated and preserved in our lab, was propagated in the grouper fin cell line (GF-1) grown in L-15 (Life Technologies, USA) containing 5% fetal bovine serum (FBS) at 28°C.

NNV-free pearl gentian grouper (*Epinephelus lanceolatus* × *Epinephelus fuscoguttatus*) juveniles (5.12 ± 0.63 g) were purchased from a commercial fish farm (Wenchang, Hainan, China). Real-time quantitative polymerase chain reaction (qPCR) was used to detect NNV in purchased fish. Primers of NNV_detection_ F: ATGGTACGCAAAGGTGAGAAG and NNV_detection_ R: TTAGTIITCCGAGTCAACCCTG were used for NNV detection. Grouper were reared in a recirculating water system with filtered seawater at 28.0 ± 1.0°C. Each tank contains 50 L seawater with continuous aeration. Fish were fed twice daily and acclimated for 15 days before experiments.

### Proteins and antibodies

The purified recombinant CP protein and pET-32a-CP plasmid were prepared and preserved in our laboratory. The purified recombinant A1, A2, A3, A4, and A5 proteins were prepared by Genecreate Co. (Wuhan). The mouse anti-grouper IgM antibody (catalog no. F05) was purchased from Aquatic Diagnostics Ltd. (England). HRP-conjugated anti-M13 antibody (catalog no. sc-53004) was purchased from Santa Cruz Biotechnology (USA). The mouse anti-His tag antibody (catalog no. AF2876) and HRP-conjugated goat anti-mouse IgG (catalog no. A0216) were purchased from Beyotime (China). The mouse anti-grouper CD4 antibody, mouse anti-grouper CD8 antibody, mouse anti-grouper IL-2, mouse anti-grouper MHC-II antibody, and mouse anti-grouper CP antibody were prepared and preserved in our lab.

### Construction of nti-NNV phage display antibody library

Groupers were intraperitoneally injected with nonlethal NNV (1 × 10^4.3^ TCID_50_/fish). On the fourth week post-challenge, the spleen of the vaccinated fish was isolated and used to extract RNA. The method of RNA isolation was used according to the procedure of simple total RNA kit (Tiangen Biotech, China).

The variable heavy (VH) and light chain (VL) regions from available pearl gentian grouper immunoglobulin sequences were utilized to design degenerate primers and construct a library. Corresponding protein sequences were sourced from the National Center for Biotechnology Information (NCBI). Following comparative analysis in MEGA7, primers targeting conserved framework regions were synthesized to build an anti-NNV phage display single-chain antibody (scFv) library. VH and VL fragments were amplified from total spleen RNA of challenged adult groupers using the primers specified in Table S1. Amplification for primer set A involved the following: initial denaturation (95°C, 3 min); 35 cycles of denaturation (95°C, 30 s), annealing (55°C, 30 s), and extension (72°C, 30 s), followed by final extension (72°C, 5 min). Target amplicons were gel-purified, and scFv fragments were assembled via primer extension PCR. The phage display vector pCantab5E (HonorGene, VSW0284), scFv fragments, and sdAb fragments underwent double digestion with NotI-HF (NEB, R3189) and SfiI (NEB, R0123). Digested scFv fragments were ligated into pCantab5E using T4 DNA ligase. Recombinant plasmids were transformed into electrocompetent TG1 super-receptor cells (Miaoling Biology, T0037) for library generation. Progeny phage production was initiated by infecting transformed TG1 cells with helper phage M13K07 (NEB, N0315S). Cell-secreted recombinant phage particles displaying the fusion protein were precipitated using PEG/NaCl solution (Solarbio, P8260). Library capacity was subsequently assessed by plate counting following primary library construction.

### Biopanning of anti-NNV phage display antibody library

Biopanning was performed by directly coating the plastic surface of a 96-well microplate with NNV target (100 mg/mL in 0.1 M NaHCO₃ coating buffer, pH 8.6), followed by overnight incubation at 4℃. Three replicates were processed per biopanning round. The next day, the coating solution was discarded, and residual liquid was removed by firmly slapping the inverted plate. Each well was then completely blocked with 5% skim milk powder in PBS for 2 h at 37°C, followed by three washes using PBST (PBS containing 0.1% [v/v] Tween 20). The prepared phage library was added to the plate (100 µL/well) and incubated for 2 h at 37°C to facilitate binding. Nonbinding phages were removed, and the wells were washed 10 times with PBST. Bound phages were subsequently eluted using 100 µL of 0.2 M glycine-HCl solution (pH 2.2), with gentle rocking for 10 min. The eluent was transferred to microcentrifuge tubes and immediately neutralized with an equal volume of 1 M Tris-HCl (pH 7.4; Solarbio, catalog no. T1090). Eluted phages were mixed with fresh TG1 culture and incubated in a 37°C water bath for 30 min and then shaken for an additional 30 min at 37°C. A 10 mL aliquot of this mixture was used to determine phage output for the round. The remaining eluent underwent phage amplification to prepare for subsequent biopanning. Three successive rounds were conducted to ensure efficient enrichment of specific phages.

### Phage-ELISA quantitative screening and DNA sequencing of positive clones

Following three biopanning rounds, phage clones were randomly selected from the final round plate for target specificity confirmation via phage-ELISA. Phage lysate preparation proceeded as follows: Individual colonies were expanded in 2 × YT Amp medium to logarithmic growth phase, co-incubated with M13K07 helper phage for 5 h, then centrifuged (5,000 rpm, 20 min). Pellets were resuspended in 2 × YT AmpKan medium and incubated for 12 h at 37°C. The resulting culture was centrifuged (10,000 rpm, 20 min, 4°C), followed by PEG/NaCl precipitation of the supernatant (5 h, 4°C). After successive centrifugation (12,000 rpm, 30 min, 4°C) and supernatant removal, pellets were resuspended in PBS and centrifuged again. The retained supernatant served as the phage source for ELISA. For phage-ELISA, microplates were coated overnight at 4°C with 100 µL/well of NNV (100 µg/mL in 0.1 M NaHCO₃, pH 8.6). After discarding unbound solution, wells were blocked completely (1 h, 37°C) and washed thrice with PBST (PBS + 0.05% [v/v] Tween-20). Following plate tapping, phage lysates were added to coated wells (three replicates per clone) and incubated (2 h, 37°C). Post-wash, M13 major coat protein antibody (RL-ph1 HRP; Santa Cruz, sc-53004) diluted 1:1,000 in blocking buffer was applied. After incubation and subsequent washes, TMB substrate (Solarbio, PR1200) was added (30 min, 37°C). Reactions were stopped with stop solution (Solarbio, C1058), and OD₄₅₀ was measured within 20 min. Clones exhibiting P/N ratios > 2.1 (P: NNV-coated well absorbance; N: negative control) were considered positive. Plasmids from expanded positive monoclonal colonies were extracted and sequenced by Sangon Biotech (Shanghai).

### Surface-plasmon resonance (SPR) analysis

Experiments were conducted at 25°C on a BIAcore 1K instrument using CM5 sensor chips. Data analysis utilized BIAcore 1K Evaluation software (Cytiva) according to the manufacturer’s protocol. Briefly, a flow cell on the CM5 chip was activated with 200 µM 1-ethyl-3-(3-dimethylaminopropyl)carbodiimide (EDC) and 50 µM N-hydroxysuccinimide (NHS) at 10 µL/min for 420 s. Subsequently, 50 µL of protein solution (prepared in 180 µL of 10 mM sodium acetate, pH 5.0) was immobilized onto the activated surface at 10 µL/min for 420 s, performed in duplicate. The cell was then blocked with 1 M ethanolamine (10 µL/min, 420 s). An adjacent reference flow cell underwent identical activation and blocking procedures but received PBS (pH 5.0) instead of protein during immobilization. Both flow cells were equilibrated with PBS prior to analysis. Molecule stock solutions were serially diluted in PBS and injected at 10 µL/min for 150 s per cycle. Following each injection, the cells were regenerated for 5 min with 10 mM glycine-HCl (pH 2.0) at 10 µL/min. Sample cell data, collected via Biacore Insight (v.2.0, Cytiva), were reference-subtracted. Association and dissociation constants were determined by globally fitting the processed data to a 1:1 Langmuir binding model using the BIAcore 1K Evaluation software (Cytiva, Marlborough, MA, USA).

### NNV inhibition assay

The NNV inhibition assay was performed as previously described (9), with some modifications. Briefly, serum from each grouper group (*n* = 10) underwent twofold serial dilution in a 96-well plate (eight replicates per dilution). Then, 50 µL of NNV (2 × 10^2^ TCID₅₀) was added to each well, thoroughly mixed, and incubated at 20℃ for 2 h. Subsequently, 100 µL of the serum–virus mixture was transferred to a new 96-well plate containing a confluent GF-1 cell monolayer and further incubated at 28°C (5% CO₂) for 2 h. After removal of the mixture, the monolayer was rinsed, and 200 µL of L-15 containing 10% FBS was added to each well. Plates were then incubated at 20°C (5% CO₂) for four days. Following the discarding of the overlay medium and three PBS washes, cells were stained with 1% crystal violet solution. Plaque formation was then assessed, and neutralizing antibody titers were calculated using the Reed-Muench method.

### Molecular docking

Three-dimensional (3D) structures of A2, CP, CP^64-83^, grouper TLRs (TLR1, TLR2, TLR3, and TLR7) were predicted by AlphaFold3. The ZDOCK server (https://zdock.wenglab.org/) was used to predict the potential binding of A2-CP, CP^64-83^-TLR1, CP^64-83^-TLR2, CP^64-83^-TLR3, and CP^64-83^-TLR7, keeping all the parameters to default values. PyMOL software (the PyMOL Molecular Graphics System, Version 2.0 Schrödinger, LLC) was used to visualize the docking results.

### CP^64-83^-Q11 nanofiber synthesis and characteristics

Q11, CP^64-83^, and CP^64-83^-Q11 peptides were synthesized and purified by Genecreate Co. (Wuhan). Q11 and CP^64-83^-Q11 peptides could self-assemble into nanofibers in the salts (pH = 7). The morphology of the CP^64-83^-Q11 was determined by transmission electron microscopy (TEM) using a Hitachi Model H-7700 microscope (Hitachi, Japan). The particle size of reassembled CP^64-83^-Q11 was determined using a Zetasizer (nano ZS90, Malvern Panalytical, UK). The secondary structure of CP^64-83^-Q11 nanovaccine was analyzed by circular dichroism (CD) spectra (Chirascan V100, Applied Photophysics, UK).

### Safety evaluation of the CP^64-83^-Q11 nanofiber

The safety evaluation of the synthesized nanofiber was assessed using *in vitro* and *in vivo* models. For the *in vitro* cytotoxicity evaluation, the standard MTT assay was employed. GF-1 cells and grouper macrophages were seeded into 96-well plates at a density of 5 × 10⁵ cells per well. These cells were then exposed to varying concentrations (1, 5, 10, 20, and 40 µg/mL) of Q11, CP^64-83^, CP, or CP^64-83^-Q11 for 24 h. Following incubation, the plates were centrifuged at 900 rpm for 3 min. After supernatant removal, MTT solution (5 mg/mL) was added. A subsequent centrifugation step was performed, the supernatant was discarded again, and DMSO was added to fully dissolve the formazan crystals. Absorbance was then measured at 490 nm using a microplate reader. Cell viability was calculated as follows: Cell viability = (OD_treatment group_ − OD_blank_)/(OD_control group_ − OD_blank_) × 100%. For the *in vivo* assessment, groupers were randomly allocated into five experimental groups (control, Q11, CP^64-83^, CP, and CP^64-83^-Q11), each containing 78 fish. Immunization was performed via intraperitoneal injection with 2 mM solutions of the respective compounds (Q11, CP^64-83^, CP, or CP^64-83^-Q11). Additionally, the initial and final weights of the fish were monitored throughout the 28-day period.

### Vaccine uptake analysis

Cellular uptake of the CP^64-83^-Q11 construct was assessed using confocal laser scanning microscopy (CLSM, LSM900, Carl Zeiss, Germany). Macrophages were plated in 6-well plates at 1 × 10⁶/mL density for 24 h, followed by 12 h of co-incubation with FITC, FITC-labeled CP^64-83^, or CP^64-83^-Q11. Nuclei were stained with Hoechst according to the manufacturer’s protocol, and uptake was qualitatively analyzed via flow cytometry. *In vivo* uptake of CP^64-83^-Q11 was evaluated by fluorescence imaging and CLSM (LSM900, Zeiss). Groupers (20 fish/group) were divided into three groups (control, CP^64-83^, and CP^64-83^-Q11) and intraperitoneally injected with 2 mM PBS, FITC-CP^64-83^, and FITC-CP^64-83^-Q11, respectively. After 6 h, the spleens were excised and analyzed using a live imaging system (AniView 100, BLT, China). Fluorescence intensity in tissues was quantified with AniView software. Spleen samples were fixed in 4% paraformaldehyde, cryosectioned (HM550 Microtome Cryostat, Leica, Germany), stained with DAPI, and examined by CLSM. Fluorescence intensity in tissue sections was quantified using ImageJ software.

### Vaccination and challenge

The vaccination procedure was conducted at room temperature. Healthy groupers received intraperitoneal injections of Q11, CP^64-83^, CP, and CP^64-83^-Q11, each administered at a dose of 2 mM. Fish injected with PBS served as the negative control. On day 28 post-vaccination (dpv), the fish were challenged with NNV via intraperitoneal injection of 100 µL containing virus at a concentration of 1 × 10^8.6^ TCID_50_/fish. Daily observations and records were maintained following the challenge.

### Enzyme-linked immunosorbent assay (ELISA) analysis

The protein expression levels of antigen-specific antibody, total antibody, CD4, CD8, IL-2, and MHC-II were quantified using ELISA, following established methods (9). For antigen-specific antibody detection, NNV-coated ELISA plates were incubated at 4°C for 12 h. After blocking with 3% BSA (37°C, 1 h), serum samples (1:50 dilution) were added as primary antibodies and incubated at 37°C for 2 h. Following PBST washes, HRP-conjugated anti-grouper IgM (1:1,000 dilution) was applied as the secondary antibody (37°C, 2 h). After that, using tetramethylbenzidine (Qiagen, Germany) as a colorimetric substrate, color was developed, followed by measurement of absorbance at 450 nm. Total antibody detection followed a similar protocol: serum (1:50) was directly coated onto plates and then blocked with 3% BSA before adding HRP-conjugated anti-grouper IgM (1:3,000). For the detection of CD4, CD8, IL-2, and MHC-II, first the sampled spleen was homogenized thoroughly by a homogenizer. Following that, it was centrifuged at 3,000 rpm for 20 min. After that, the supernatant was collected for use. Mouse anti-grouper CD4, CD8, IL-2, and MHC-II polyclonal antibodies (diluted 1:1000) were used as the primary antibodies, while HRP-conjugated goat anti-mouse IgG (diluted 1:3,000) was used as the secondary antibody.

### Quantitative real-time polymerase chain reaction (qPCR) analysis

The copy number of NNV in the brain and eye was detected by qPCR analysis. RNA was extracted from homogenized tissue using TRIZOL (Tiangen Biotech, China), according to the manufacturer’s instructions. The concentration of RNA was measured by Nanodrop 8000 Spectrophotometer (Pierce/Thermo Scientific, Waltham, MA, USA) and reverse transcribed into cDNA using HiScript QRT SuperMix for qPCR kit (Vazyme, China) following the manufacturer’s instructions. The cDNA was used as a template for qPCR using AceQ qPCR SYBR Green Master Mix (Vazyme, China) in a CFX96 Real-Time PCR Detection System (Bio-Rad, USA). Each analysis was repeated three times, and *β-actin* (Accession number: AY510710) was used as the internal reference gene. The relative expression was calculated using 2^-ΔΔct^ method.

## Data Availability

All data supporting the findings of this study are available in the article and supplemental material.

## SUPPLEMENTAL MATERIAL

Supplemental material jvi.00319-25-s0001.doc Figures S1 to S9; Tables S1 to S3.

ASM does not own the copyrights to Supplemental Material that may be linked to, or accessed through, an article. The authors have granted ASM a non-exclusive, world-wide license to publish the Supplemental Material files. Please contact the corresponding author directly for reuse.

## References

[1] Strumillo ST, Kartavykh D, de Carvalho FF Jr, Cruz NC, de Souza Teodoro AC, Sobhie Diaz R, Curcio MF. 2021. Host-virus interaction and viral evasion. Cell Biol Int 45:1124–1147. doi:10.1002/cbin.11565 33533523 PMC8014853

[2] Chudnovets A, Liu J, Narasimhan H, Liu Y, Burd I. 2020. Role of inflammation in virus pathogenesis during pregnancy. J Virol 95:e01381–01319. doi:10.1128/JVI.01381-19 33115865 PMC7944452

[3] Vázquez-Salgado L, Olveira JG, Bandín I. 2022. Nervous necrosis virus viability modulation by water salinity and temperature. J Fish Dis 45:561–568. doi:10.1111/jfd.13581 35007369

[4] Liu J, Wang L, Zhang X, Wang S, Qin Q. 2024. Nervous necrosis virus induced vacuolization is a Rab5- and actin-dependent process. Virulence 15:2301244. doi:10.1080/21505594.2023.2301244 38230744 PMC10795790

[5] Padrós F, Caggiano M, Toffan A, Constenla M, Zarza C, Ciulli S. 2022. Integrated management strategies for viral nervous necrosis (VNN) disease control in marine fish farming in the Mediterranean. Pathogens 11:330. doi:10.3390/pathogens11030330 35335654 PMC8955002

[6] Toubanaki DK, Efstathiou A, Karagouni E. 2022. Transcriptomic analysis of fish hosts responses to nervous necrosis virus. Pathogens 11:201. doi:10.3390/pathogens11020201 35215144 PMC8875540

[7] Leiva-Rebollo R, Labella AM, Gémez-Mata J, Castro D, Borrego JJ. 2024. Fish Iridoviridae: infection, vaccination and immune response. Vet Res 55:88. doi:10.1186/s13567-024-01347-1 39010235 PMC11247874

[8] Zhang Y, Dong F, Xing J, Tang X, Sheng X, Chi H, Zhan W. 2022. Characterization of nervous necrosis virus (NNV) nonstructural protein B2 and its enhancement on virus proliferation. Viruses 14:2818. doi:10.3390/v14122818 36560822 PMC9786564

[9] Lin T, Xing J, Tang X, Sheng XZ, Chi H, Zhan WB. 2023. Immune and protective effects of subunit vaccines from S-domain or P-domain in capsid protein against nervous necrosis virus in pearl gentian grouper. Aquaculture 566:739177. doi:10.1016/j.aquaculture.2022.739177

[10] Kamath SD, Bublin M, Kitamura K, Matsui T, Ito K, Lopata AL. 2023. Cross-reactive epitopes and their role in food allergy. J Allergy Clin Immunol 151:1178–1190. doi:10.1016/j.jaci.2022.12.827 36932025

[11] Liu J, Wang Z, Ma J, Ji S, Huo Y. 2024. Identification of a norovirus GII-specific antigenic epitope. Arch Virol 169:131. doi:10.1007/s00705-024-06060-0 38819530

[12] Lin CF, Jiang HK, Chen NC, Wang TY, Chen TY. 2018. Novel subunit vaccine with linear array epitope protect giant grouper against nervous necrosis virus infection. Fish Shellfish Immunol 74:551–558. doi:10.1016/j.fsi.2018.01.029 29355759

[13] Ledsgaard L, Ljungars A, Rimbault C, Sørensen CV, Tulika T, Wade J, Wouters Y, McCafferty J, Laustsen AH. 2022. Advances in antibody phage display technology. Drug Discov Today 27:2151–2169. doi:10.1016/j.drudis.2022.05.002 35550436

[14] Zhang K, Tang Y, Chen Q, Liu Y. 2022. The screening of therapeutic peptides for anti-Inflammation through phage display technology. Int J Mol Sci 23:8554. doi:10.3390/ijms23158554 35955688 PMC9368796

[15] Nagano K, Tsutsumi Y. 2021. Phage display technology as a powerful platform for antibody drug discovery. Viruses 13:178. doi:10.3390/v13020178 33504115 PMC7912188

[16] Jaroszewicz W, Morcinek-Orłowska J, Pierzynowska K, Gaffke L, Węgrzyn G. 2022. Phage display and other peptide display technologies. FEMS Microbiol Rev 46:fuab052. doi:10.1093/femsre/fuab052 34673942

[17] Hutchings CJ, Sato AK. 2024. Phage display technology and its impact in the discovery of novel protein-based drugs. Expert Opin Drug Discov 19:887–915. doi:10.1080/17460441.2024.2367023 39074492

[18] Takakusagi Y, Takakusagi K, Sakaguchi K, Sugawara F. 2020. Phage display technology for target determination of small-molecule therapeutics: an update. Expert Opin Drug Discov 15:1199–1211. doi:10.1080/17460441.2020.1790523 32660284

[19] França RKA, Studart IC, Bezerra MRL, Pontes LQ, Barbosa AMA, Brigido MM, Furtado GP, Maranhão AQ. 2023. Progress on phage display technology: tailoring antibodies for cancer immunotherapy. Viruses 15:1903. doi:10.3390/v15091903 37766309 PMC10536222

[20] Zhang Y. 2023. Evolution of phage display libraries for therapeutic antibody discovery. MAbs 15:2213793. doi:10.1080/19420862.2023.2213793 37222232 PMC10210849

[21] Mustafa MI, Mohammed A. 2024. Developing recombinant antibodies by phage display technology to neutralize viral infectious diseases. SLAS Discov 29:100140. doi:10.1016/j.slasd.2024.01.001 38182043

[22] Desai DN, Mahal A, Varshney R, Obaidullah AJ, Gupta B, Mohanty P, Pattnaik P, Mohapatra NC, Mishra S, Kandi V, Rabaan AA, Mohapatra RK. 2023. Nanoadjuvants: promising bioinspired and biomimetic approaches in vaccine innovation. ACS Omega 8:27953–27968. doi:10.1021/acsomega.3c02030 37576639 PMC10413842

[23] Morales-Becerril A, Aranda-Lara L, Isaac-Olivé K, Ocampo-García BE, Morales-Ávila E. 2022. Nanocarriers for delivery of siRNA as gene silencing mediator. EXCLI J 21:1028–1052. doi:10.17179/excli2022-4975 36110562 PMC9441682

[24] Hill A, Beitelshees M, Pfeifer BA. 2021. Vaccine delivery and immune response basics. Methods Mol Biol 2183:1–8. doi:10.1007/978-1-0716-0795-4_1 32959236

[25] Das A, Ali N. 2021. Nanovaccine: an emerging strategy. Expert Rev Vaccines 20:1273–1290. doi:10.1080/14760584.2021.1984890 34550859

[26] Mansoor I, Eassa HA, Mohammed KHA, Abd El-Fattah MA, Abdo MH, Rashad E, Eassa HA, Saleh A, Amin OM, Nounou MI, Ghoneim O. 2022. Microneedle-based vaccine delivery: review of an emerging technology. AAPS PharmSciTech 23:103. doi:10.1208/s12249-022-02250-8 35381906 PMC8982652

[27] Liu G, Zhu M, Zhao X, Nie G. 2021. Nanotechnology-empowered vaccine delivery for enhancing CD8^+^ T cells-mediated cellular immunity. Adv Drug Deliv Rev 176:113889. doi:10.1016/j.addr.2021.113889 34364931

[28] Zeng C, Zhang C, Walker PG, Dong Y. 2022. Formulation and delivery technologies for mRNA vaccines. Curr Top Microbiol Immunol 440:71–110. doi:10.1007/82_2020_217 32483657 PMC8195316

[29] He A, Li X, Dai Z, Li Q, Zhang Y, Ding M, Wen ZF, Mou Y, Dong H. 2023. Nanovaccine-based strategies for lymph node targeted delivery and imaging in tumor immunotherapy. J Nanobiotechnology 21:236. doi:10.1186/s12951-023-01989-x 37482608 PMC10364424

[30] Lee J, Khang D. 2022. Mucosal delivery of nanovaccine strategy against COVID-19 and its variants. Acta Pharm Sin B 13:2897–2925. doi:10.1016/j.apsb.2022.11.022 36438851 PMC9676163

[31] Pan C, Ye J, Zhang S, Li X, Shi Y, Guo Y, Wang K, Sun P, Wu J, Wang H, Zhu L. 2023. Production of a promising modular proteinaceous self-assembled delivery system for vaccination. Nanoscale 15:10794–10807. doi:10.1039/d2nr06718h 37326289

[32] Zhao X, Zhao R, Nie G. 2022. Nanocarriers based on bacterial membrane materials for cancer vaccine delivery. Nat Protoc 17:2240–2274. doi:10.1038/s41596-022-00713-7 35879454

[33] Hinke DM, Andersen TK, Gopalakrishnan RP, Skullerud LM, Werninghaus IC, Grødeland G, Fossum E, Braathen R, Bogen B. 2022. Antigen bivalency of antigen-presenting cell-targeted vaccines increases B cell responses. Cell Rep 39:110901. doi:10.1016/j.celrep.2022.110901 35649357

[34] Rudra JS, Tian YF, Jung JP, Collier JH. 2010. A self-assembling peptide acting as an immune adjuvant. Proc Natl Acad Sci USA 107:622–627. doi:10.1073/pnas.0912124107 20080728 PMC2818904

[35] Rudra JS, Mishra S, Chong AS, Mitchell RA, Nardin EH, Nussenzweig V, Collier JH. 2012. Self-assembled peptide nanofibers raising durable antibody responses against a malaria epitope. Biomaterials 33:6476–6484. doi:10.1016/j.biomaterials.2012.05.041 22695068 PMC3392361

[36] Hainline KM, Haddad HF, Gilpin A, Curvino EJ, Varghese S, Collier JH. 2024. Active immunotherapy for C5a-mediated inflammation using adjuvant-free self-assembled peptide nanofibers. Acta Biomater 179:83–94. doi:10.1016/j.actbio.2024.02.042 38447809 PMC11045302

[37] Wu Y, Kelly SH, Sanchez-Perez L, Sampson JH, Collier JH. 2020. Comparative study of α-helical and β-sheet self-assembled peptide nanofiber vaccine platforms: influence of integrated T-cell epitopes. Biomater Sci 8:3522–3535. doi:10.1039/d0bm00521e 32452474 PMC7665831

[38] Zhang B, Yang H, Cai G, Nie Q, Sun Y. 2024. The interactions between the host immunity and intestinal microorganisms in fish. Appl Microbiol Biotechnol 108:30. doi:10.1007/s00253-023-12934-1 38170313 PMC13497495

[39] Salinas I, Fernández-Montero Á, Ding Y, Sunyer JO. 2021. Mucosal immunoglobulins of teleost fish: a decade of advances. Dev Comp Immunol 121:104079. doi:10.1016/j.dci.2021.104079 33785432 PMC8177558

[40] Yu Y, Wang Q, Huang Z, Ding L, Xu Z. 2020. Immunoglobulins, mucosal immunity and vaccination in teleost fish. Front Immunol 11:567941. doi:10.3389/fimmu.2020.567941 33123139 PMC7566178

[41] Bilal S, Etayo A, Hordvik I. 2021. Immunoglobulins in teleosts. Immunogenetics 73:65–77. doi:10.1007/s00251-020-01195-1 33439286

[42] Yeoh SG, Sum JS, Lai JY, W Isa WYH, Lim TS. 2022. Potential of phage display antibody technology for cardiovascular disease immunotherapy. J Cardiovasc Transl Res 15:360–380. doi:10.1007/s12265-021-10169-x 34467463

[43] Pung HS, Tye GJ, Leow CH, Ng WK, Lai NS. 2023. Generation of peptides using phage display technology for cancer diagnosis and molecular imaging. Mol Biol Rep 50:4653–4664. doi:10.1007/s11033-023-08380-x 37014570 PMC10072011

[44] Nur A, Schubert M, Lai JY, Hust M, Choong YS, Isa W, Lim TS. 2023. Antibody phage display. Methods Mol Biol 2702:3–12. doi:10.1007/978-1-0716-3381-6_1 37679612

[45] Qi H, Ma M, Lai D, Tao SC. 2021. Phage display: an ideal platform for coupling protein to nucleic acid. Acta Biochim Biophys Sin (Shanghai) 53:389–399. doi:10.1093/abbs/gmab006 33537750

[46] Yang F, Liu L, Neuenschwander PF, Idell S, Vankayalapati R, Jain KG, Du K, Ji H, Yi G. 2022. Phage display-derived peptide for the specific binding of SARS-CoV-2. ACS Omega 7:3203–3211. doi:10.1021/acsomega.1c04873 35128233 PMC8751651

[47] Qiang M, Ma P, Li Y, Liu H, Harding A, Min C, Wang F, Liu L, Yuan M, Ji Q, et al.. 2022. Neutralizing antibodies to SARS-CoV-2 selected from a human antibody library constructed decades ago. Adv Sci (Weinh) 9:e2102181. doi:10.1002/advs.202102181 34716683 PMC8646600

[48] Krohn S, Holtrop T, Brandsma AM, Moerer P, Nederend M, Darzentas N, Brüggemann M, Klausz K, Leusen JHW, Peipp M. 2024. Combining cellular immunization and phage display screening results in novel, FcγRI-specific antibodies. Viruses 16:596. doi:10.3390/v16040596 38675937 PMC11053525

[49] Zheng X, Liu Q, Liang Y, Feng W, Yu H, Tong C, Song B. 2024. Advancement in the development of single chain antibodies using phage display technology. PeerJ 12:e17143. doi:10.7717/peerj.17143 38618563 PMC11015834

[50] Mejias-Gomez O, Braghetto M, Sørensen MKD, Madsen AV, Guiu LS, Kristensen P, Pedersen LE, Goletz S. 2024. Deep mining of antibody phage-display selections using Oxford Nanopore Technologies and dual unique molecular identifiers. N Biotechnol 80:56–68. doi:10.1016/j.nbt.2024.02.001 38354946

[51] Anand T, Virmani N, Bera BC, Vaid RK, Vashisth M, Bardajatya P, Kumar A, Tripathi BN. 2021. Phage display technique as a tool for diagnosis and antibody selection for coronaviruses. Curr Microbiol 78:1124–1134. doi:10.1007/s00284-021-02398-9 33687511 PMC7941128

[52] Kelly SH, Cossette BJ, Varadhan AK, Wu Y, Collier JH. 2021. Titrating polyarginine into nanofibers enhances cyclic-dinucleotide adjuvanticity in vitro and after sublingual immunization. ACS Biomater Sci Eng 7:1876–1888. doi:10.1021/acsbiomaterials.0c01429 33775089 PMC8822437

[53] Robang AS, Wong KM, Leisen J, Liu R, Radford WL, Rao Sudarshan T, Hudalla GA, Paravastu AK. 2024. Parallel β-sheet structure and structural heterogeneity detected within Q11 self-assembling peptide nanofibers. J Phys Chem B 128:5387–5396. doi:10.1021/acs.jpcb.4c00825 38787393 PMC11163420

[54] Shetty S, Wu Y, Lloyd CZ, Mehta N, Liu Y, Woodruff ME, Segura T, Collier JH. 2025. Anti-cytokine active immunotherapy based on supramolecular peptides for alleviating IL-1β-mediated inflammation. Adv Healthc Mater 14:e2401444. doi:10.1002/adhm.202401444 39113323 PMC11802897

[55] Yang Y, Zheng P, Duan B, Yang Y, Zheng X, Li W, Liu Q, Hu Y, Ma Y. 2024. A personalized vaccine combining immunogenic cell death-induced cells and nanosized antigens for enhanced antitumor immunity. J Control Release 376:1271–1287. doi:10.1016/j.jconrel.2024.10.060 39515613

[56] Tang M, Cai JH, Diao HY, Guo WM, Yang X, Xing S. 2022. The progress of peptide vaccine clinical trials in gynecologic oncology. Hum Vaccin Immunother 18:2062982. doi:10.1080/21645515.2022.2062982 35687860 PMC9450897

[57] Nelde A, Rammensee HG, Walz JS. 2021. The peptide vaccine of the future. Mol Cell Proteomics 20:100022. doi:10.1074/mcp.R120.002309 33583769 PMC7950068

[58] O’Neill CL, Shrimali PC, Clapacs ZE, Files MA, Rudra JS. 2021. Peptide-based supramolecular vaccine systems. Acta Biomater 133:153–167. doi:10.1016/j.actbio.2021.05.003 34010691 PMC8497425

[59] Ghosh P, Bhattacharya M, Patra P, Sharma G, Patra BC, Lee SS, Sharma AR, Chakraborty C. 2022. Evaluation and designing of epitopic-peptide vaccine against Bunyamwera orthobunyavirus using M-polyprotein target sequences. Int J Pept Res Ther 28:5. doi:10.1007/s10989-021-10322-9 34867129 PMC8634745

[60] Li M, Wang H, Tian L, Pang Z, Yang Q, Huang T, Fan J, Song L, Tong Y, Fan H. 2022. COVID-19 vaccine development: milestones, lessons and prospects. Signal Transduct Target Ther 7:146. doi:10.1038/s41392-022-00996-y 35504917 PMC9062866

