# Supplementary material for: Rationally designed self-assembled peptide nanofibers provoke robust humoral immunity against nervous necrosis virus
Source: J Virol. 2025 Jul 15;99(8):e00319-25. doi: 10.1128/jvi.00319-25 (PMC12363211; doi:10.1128/jvi.00319-25)
Supplement: Supplemental material — Figures S1 to S9; Tables S1 to S3. [file jvi.00319-25-s0001.doc]

**Supplementary Information For**

**Rationally designed** **self-assembled peptide nanofibers provoke robust humoral immunity against nervous necrosis virus**

Chen Zhang, Yong-Can Zhou, Wen-Ye Song, Xin-Xin Liu, Hai-Hua Peng, Yun Sun*

School of Marine Biology and Fisheries, Sanya Institute of Breeding and Multiplication, Collaborative Innovation Center of Marine Science and Technology, Hainan University, China

*Corresponding author at: School of Marine Biology and Fisheries, Sanya Institute of Breeding and Multiplication, Collaborative Innovation Center of Marine Science and Technology, Hainan University, Haikou 570228, China

Chen Zhang and Yong-Can Zhou contributed equally to this work.

E-mail address: syshui207@126.com (Sun Yun).

**Contents:**

Supplementary figures 9

Supplementary tables 3

**Supplementary figures**


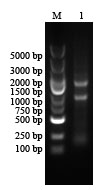


**Figure S1:** Gel electrophoresis results of grouper total RNA. M: DNA marker; lane 1: grouper total RNA.


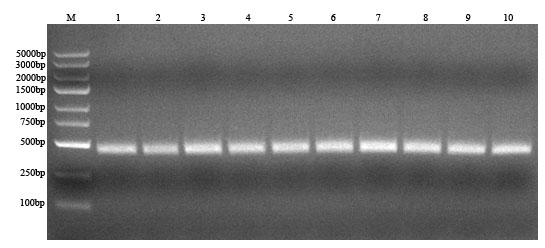

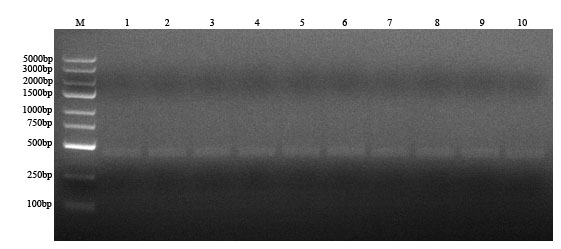


**A**

**B**

**Figure S2:** *V_H_* gene (A) and *V_L_* gene (B) amplification product using nested PCR. M: DNA marker; Lane 1-10: Product from the first step PCR.


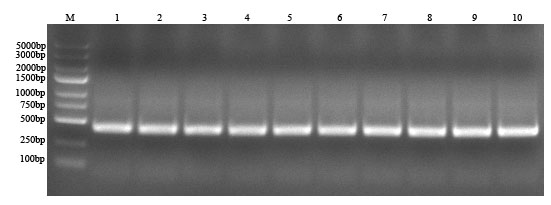

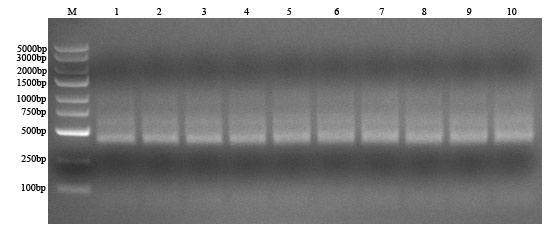


**A**

**B**

**Figure S3:** *V_H_* gene (A) and *V_L_* gene (B) amplification product using nested PCR. M: DNA marker; Lane 1-10: Product from the third step PCR.


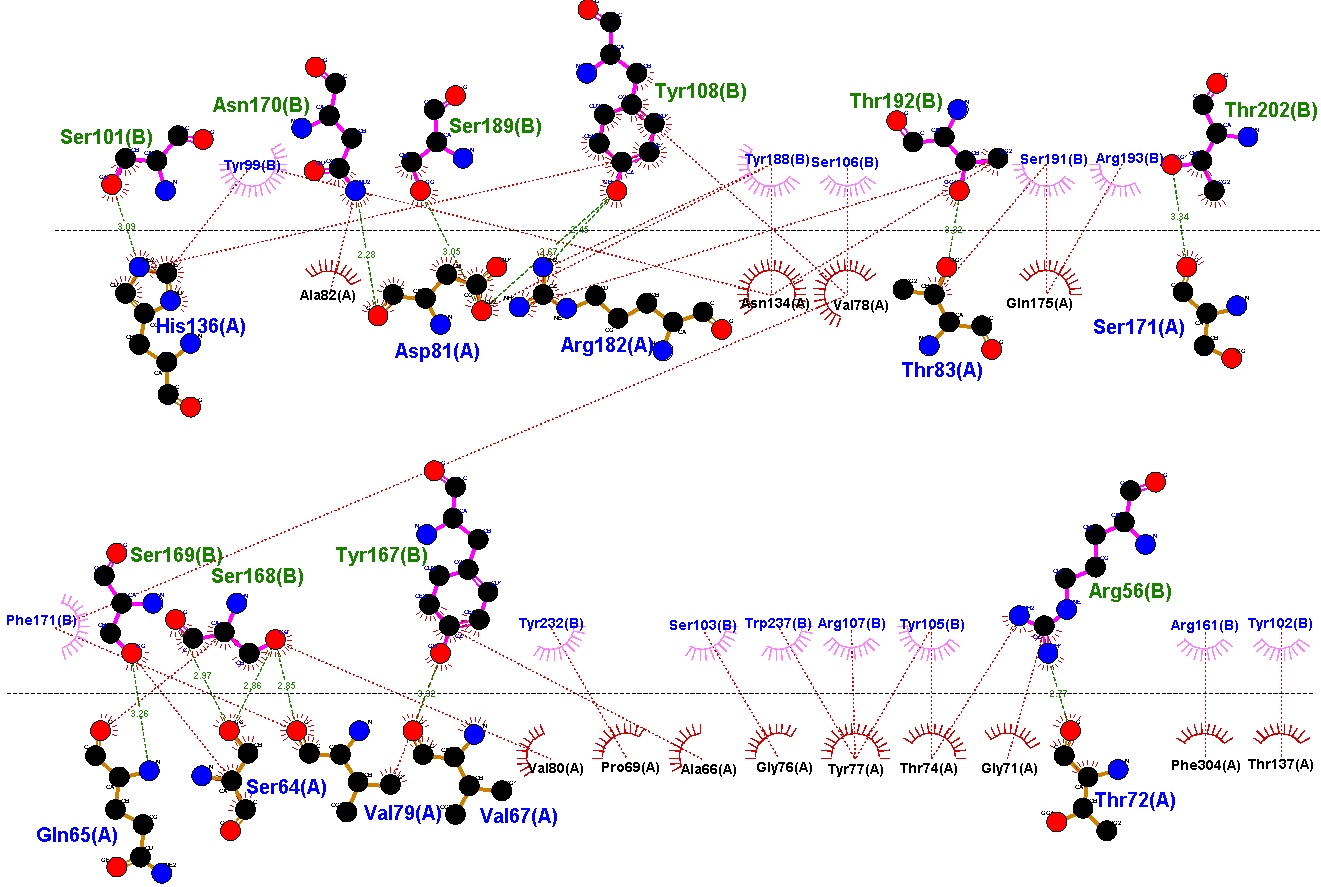


**Figure S4:** The 2D interaction diagram between A2 and CP protein.


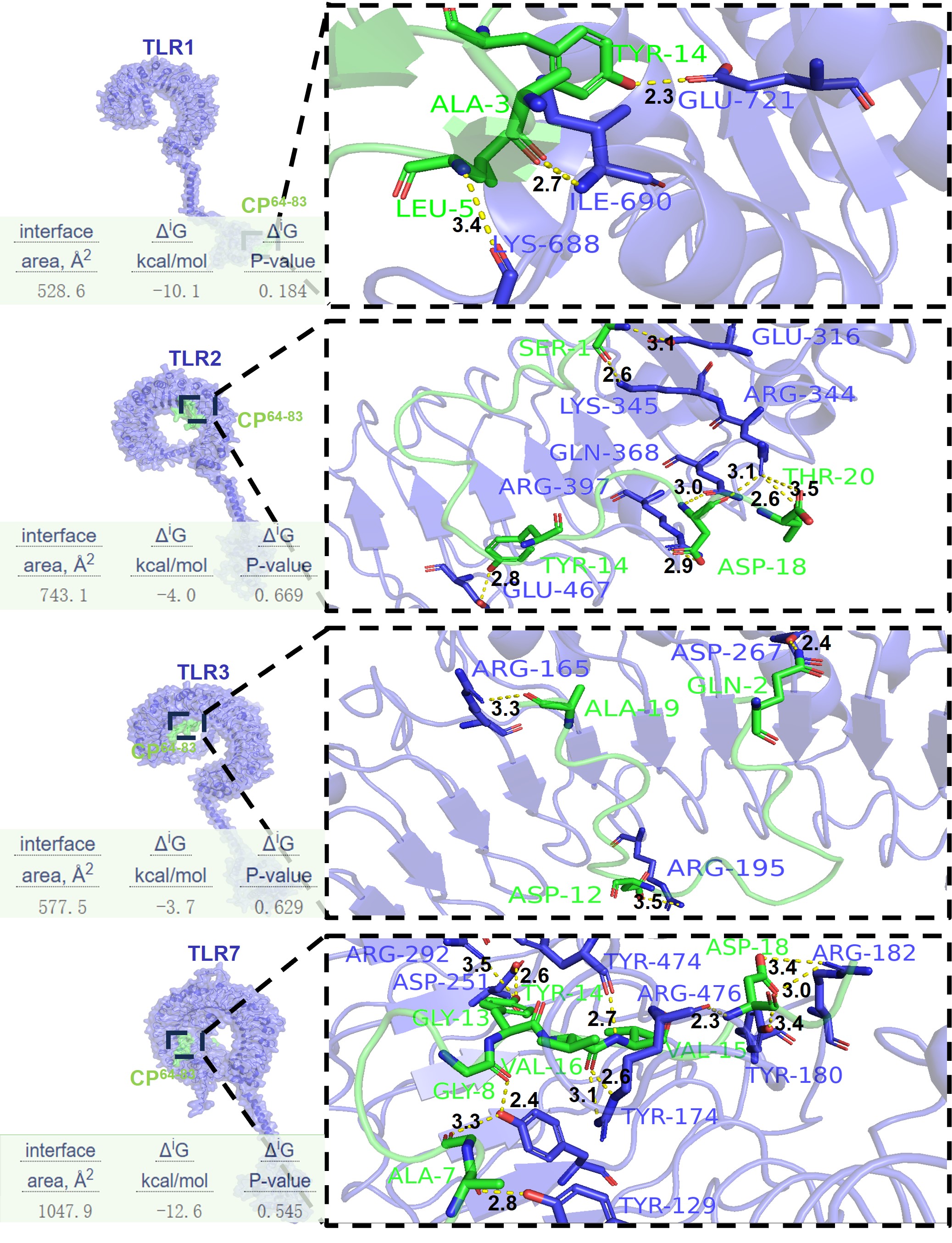


**Figure S5:** The interaction of CP^64-83^ with several TLRs. CP^64-83^ is shown in green, TLRs is shown in purple. Close-up views of the CP^64-83^-occupied sites in TLRs are displayed. The green ribbons represent CP^64-83^, the purple ribbons represent TLRs, and the hydrogen bonds are shown as yellow dashes.

**
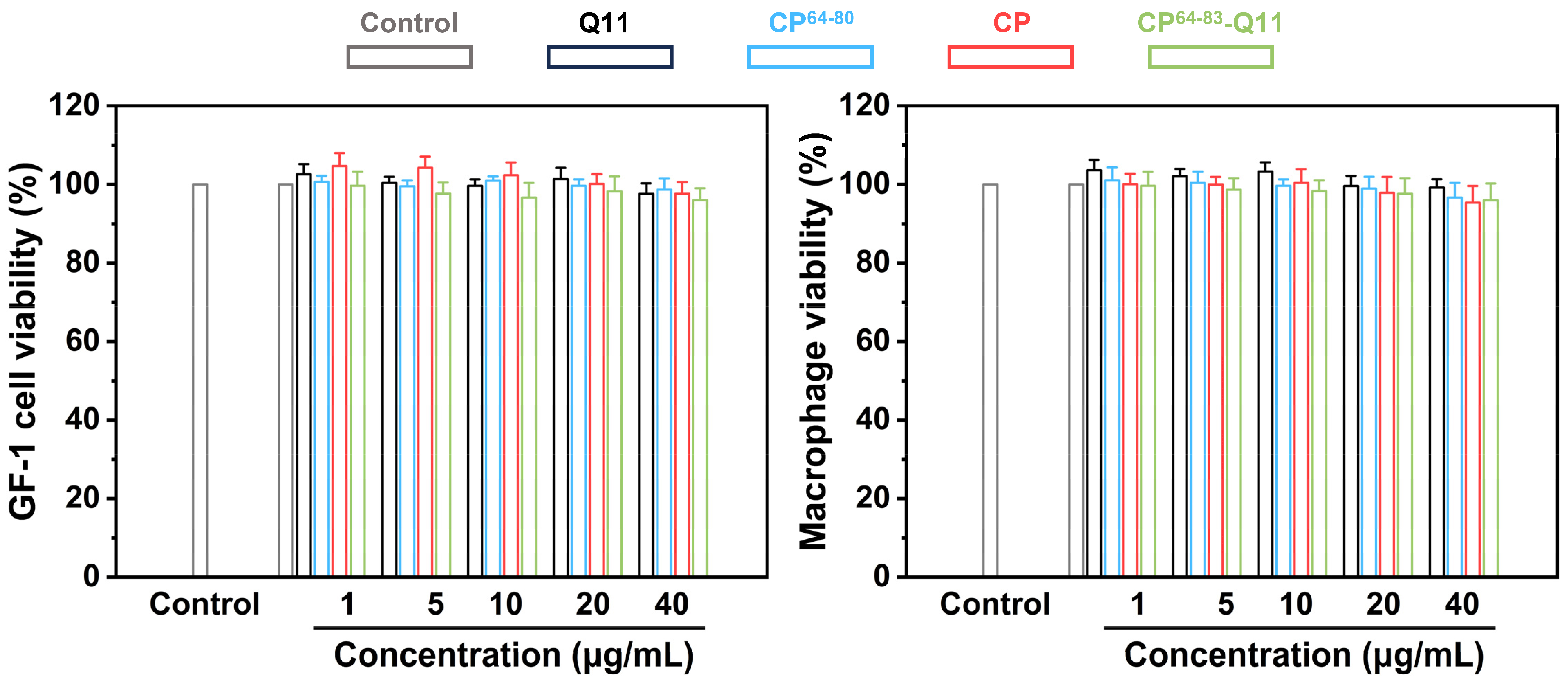
**

**Figure S6:** Cell viability of GF-1 cell and macrophage after incubation with different concentrations of Q11, CP^64-83^, CP, and CP^64-83^-Q11 for 24 h.

**
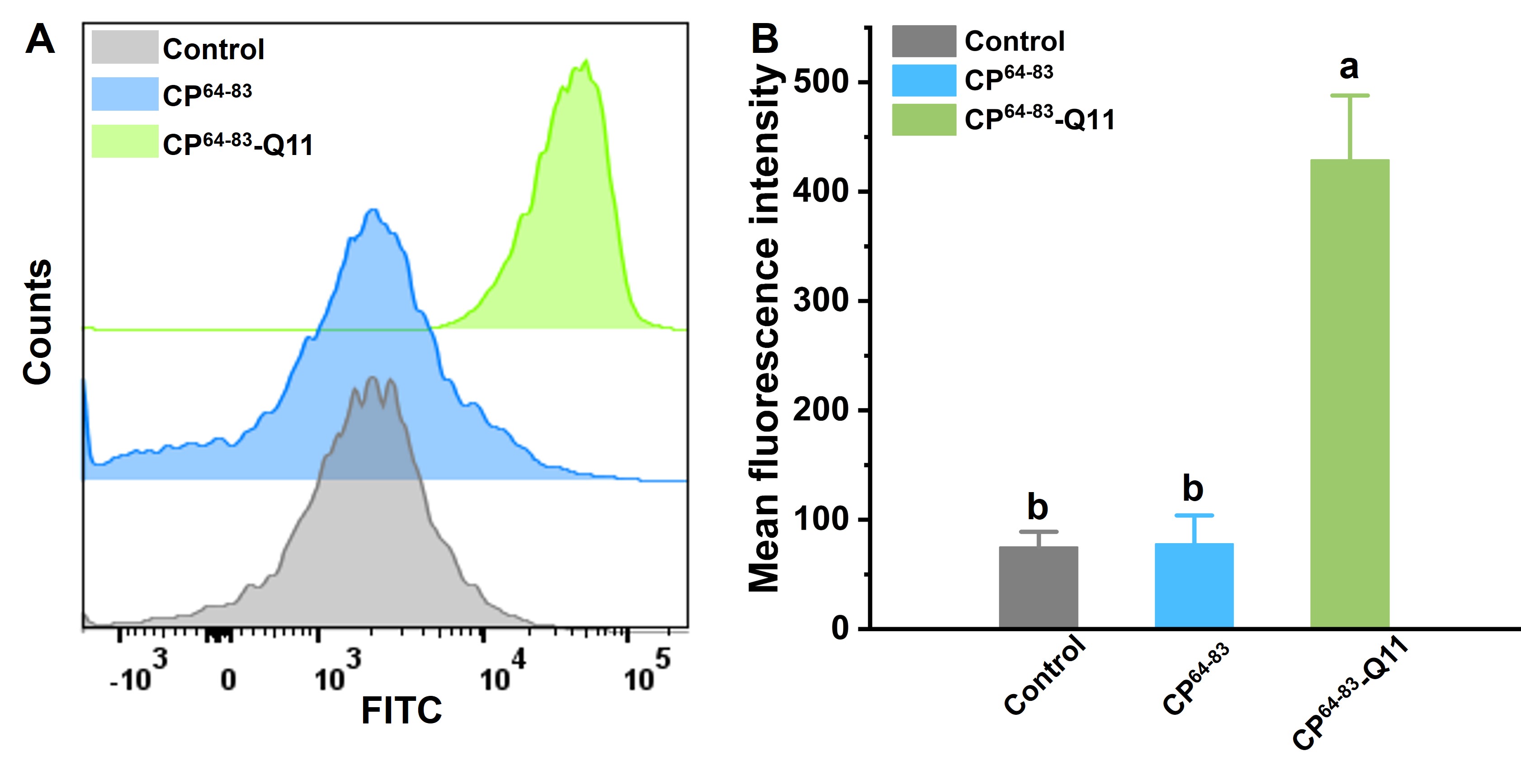
**

**Figure S7:** Flow cytometry data (A) and quantification of nanofibers cellular uptake (B) for GF-1 after incubation with FITC-labelled CP^64-83^-Q11. Data at the same sampling time with different lowercase letters (a, b, and c) are significantly different (*P* < 0.05).

**
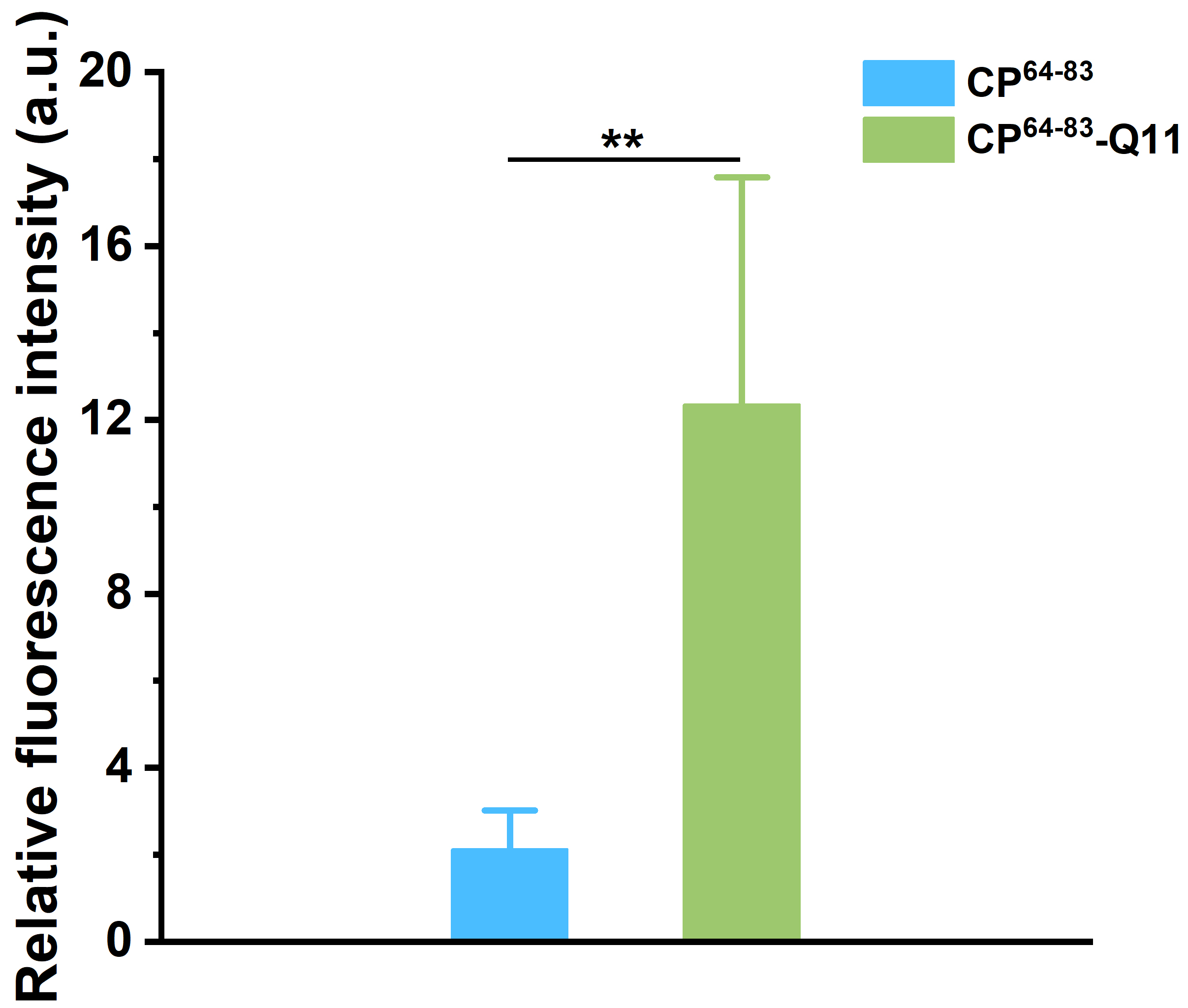
**

**Figure S8:** Quantitative fluorescence signals at the spleen based on *in vivo* imaging data shown in Figure 4B. *P* values were calculated by Student's t test ***P*<0.01, **P*<0.05).

**
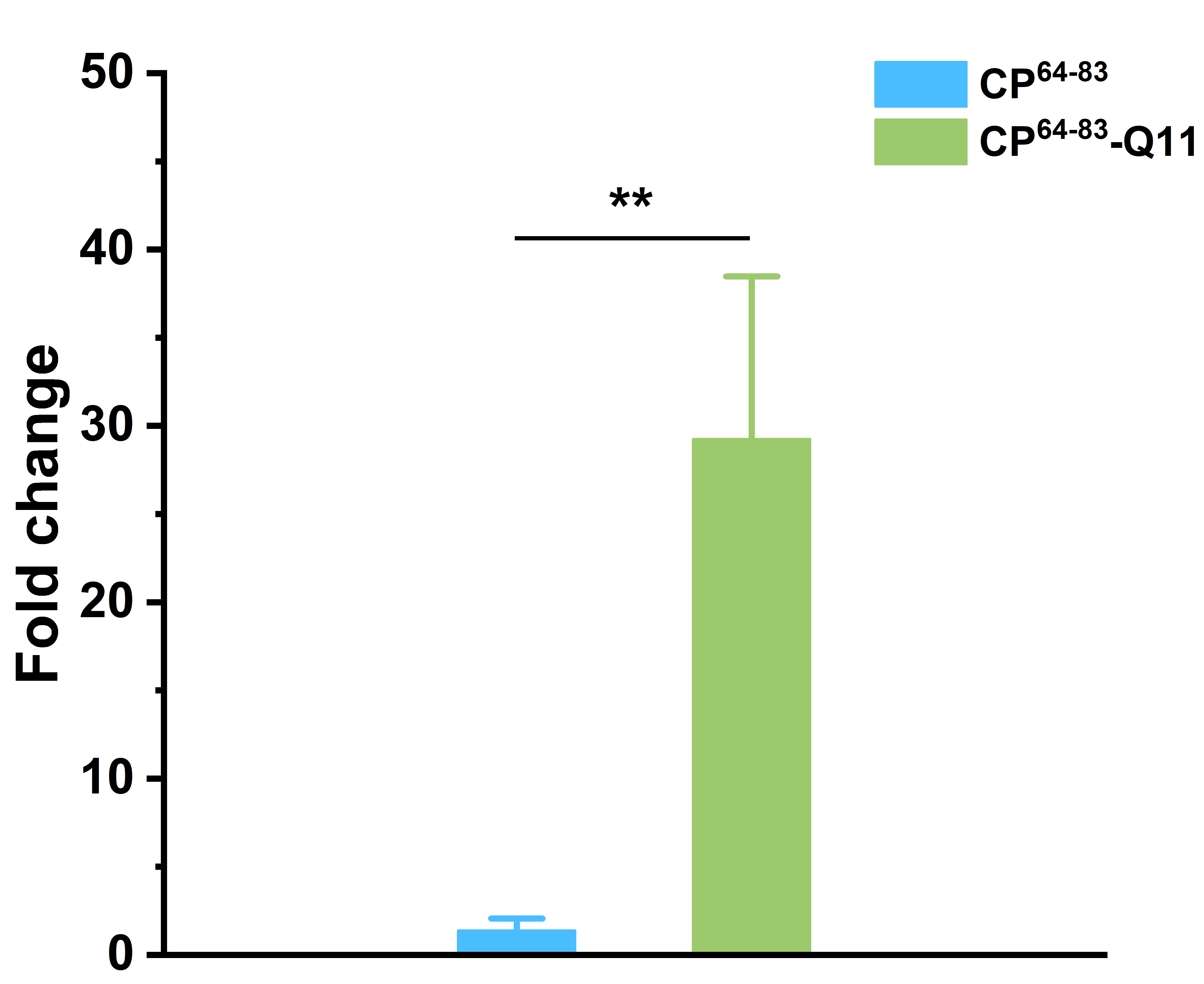
**

**Figure S9:** Quantitative fluorescence signals at the spleen analyzed by imageJ software. *P* values were calculated by Student's t test ***P*<0.01, **P*<0.05).

**Supplementary tables**

**Table S1: Primers used in this study**

| Primer names | Sequence Primer sequences (from 5′ to 3′) |
| --- | --- |
| Grouper-VH-F1 | GGACTGCTGCTTTTGACTATC |
| Grouper-VH-R1 | ACATTGCATCAGAGGAAACAC |
| Grouper-VH-F2-1 | CGTCGCGGCCCAGGCGGCCGAACAGTTGACACAGCCAGC |
| Grouper-VH-F2-2 | CGTCGCGGCCCAGGCGGCCGGTCAGACTCTG |
| Grouper-VH-F2-3 | CGTCGCGGCCCAGGCGGCCGGTCAACGTCTG |
| Grouper-VH-R2-1 | AGAACCACCTCCGCCTGAACCGCCTCCACCCGAGACTGTCACGGTTGTTCCCT |
| Grouper-VH-R2-2 | AGAACCACCTCCGCCTGAACCGCCTCCACCCGATACTGTGACCGCAGTCCCC |
| Grouper-VH-R2-3 | AGAACCACCTCCGCCTGAACCGCCTCCACCCGAGACGGTGACCGTGGTGCCT |
| Grouper-Vk-F1 | CTCATCTGGACTCTCCTCTG |
| Grouper-Vk-R1 | GGGGGGAGGACGGTCAGGGT |
| Grouper-Vk-F2 | GTTCAGGCGGAGGTGGTTCTGGCGGTGGCGGATCGCAGGTGACTGTGACTCAGCC |
| Grouper-Vk-R2-1 | ACTAATAGGCCGGCCTGGCCGTCAACGACGAGTTTGGT |
| Grouper-Vk-R2-2 | ACTAATAGGCCGGCCTGGCCACTTCCAACATCCAGTCTGGT |
| Grouper-OE-F | CGTCGCGGCCCAGCCGGCCG |
| Grouper-OE-R | ACTAATAGGCCGGCCTGGCC |
| M13-R | AGCGGATAACAATTTCACACAGGA |
| pHEN-R | GCCCCATTCAGATCCTCTTC |
| T7 promoter | TAATACGACTCACTATAGGG |
| T7 terminator | GCTAGTTATTGCTCAGCGG |
| β-actin-F | GCCCCACCAGAGCGTAAATA |
| β-actin-R | CATCGTACTCCTGCTTGCTGAT |

**Table S2:** Related parameters in the biopanning process

| Round | Phage input(cfu) | Phage Output (cfu) | Recovery efficiency | Enrichment degree |
| --- | --- | --- | --- | --- |
| 1 | 2.00×10^11^ | 2.00×10^5^ | 1.00×10^-6^ | - |
| 2 | 1.00×10^11^ | 1.54×10^5^ | 1.54×10^-6^ | 1.54 |
| 3 | 1.00×10^11^ | 1.23×10^6^ | 1.23×10^-5^ | 0.79 |

**Table S3:** The survival rate and growth performance of vaccinated grouper

| Group | Survival rate (%) | Initial weight (g) | Final weight (g) |
| --- | --- | --- | --- |
| Control | 100.00 ± 0.00^a^ | 5.12 ± 0.63 | 8.96 ± 1.02^a^ |
| Q11 | 98.26 ± 3.37^a^ | 5.12 ± 0.63 | 8.72 ± 0.56^a^ |
| CP^64-83^ | 100.00 ± 0.00^a^ | 5.12 ± 0.63 | 8.53 ± 0.73^a^ |
| CP | 96.08 ± 4.54^a^ | 5.12 ± 0.63 | 8.60 ± 0.36^a^ |
| CP^64-83^-Q11 | 98.69 ± 3.09^a^ | 5.12 ± 0.63 | 8.58 ± 0.68^a^ |
